# *OLOGRAM-MODL*: mining enriched *n*-wise combinations of genomic features with Monte Carlo and dictionary learning

**DOI:** 10.1093/nargab/lqab114

**Published:** 2021-12-22

**Authors:** Quentin Ferré, Cécile Capponi, Denis Puthier

**Affiliations:** Aix Marseille Univ, INSERM, UMR U1090, TAGC, Marseille, France; Aix Marseille Univ, CNRS, UMR 7020, LIS, Qarma, Marseille, France; Aix Marseille Univ, CNRS, UMR 7020, LIS, Qarma, Marseille, France; Aix Marseille Univ, INSERM, UMR U1090, TAGC, Marseille, France; MarMaRa Institute, TGML, 13288 Marseille, France

## Abstract

Most epigenetic marks, such as Transcriptional Regulators or histone marks, are biological objects known to work together in *n*-wise complexes. A suitable way to infer such functional associations between them is to study the overlaps of the corresponding genomic regions. However, the problem of the statistical significance of *n*-wise overlaps of genomic features is seldom tackled, which prevent rigorous studies of *n*-wise interactions. We introduce *OLOGRAM-MODL*, which considers overlaps between *n* ≥ 2 sets of genomic regions, and computes their statistical mutual enrichment by Monte Carlo fitting of a Negative Binomial distribution, resulting in more resolutive *P*-values. An optional machine learning method is proposed to find complexes of interest, using a new itemset mining algorithm based on dictionary learning which is resistant to noise inherent to biological assays. The overall approach is implemented through an easy-to-use CLI interface for workflow integration, and a visual tree-based representation of the results suited for explicability. The viability of the method is experimentally studied using both artificial and biological data. This approach is accessible through the command line interface of the *pygtftk* toolkit, available on Bioconda and from https://github.com/dputhier/pygtftk

## INTRODUCTION

Modern genomic analysis methods can localize many different types of genomic features, such as histone modifications, transcriptional regulator binding sites or gene promoters. As such, a fundamental question arises: do those *sets* of features have a functional association? A typical approach is to represent such features as regions, or intervals (hence, as ‘Browser Extensible Data’ or BED files ) and look for significant co-localization through the statistical significance of the amount of overlap between them, against (*H*_0_) of overlapping no more than by chance. This is especially important since co-localization is often associated to functional association in genomic elements (1).

Pairwise overlaps between two sets can be analyzed with methods such as *GeometriCorr*, *BEDTOOLS fisher* (2), *GREAT*, *Genomic HyperBrowser* (3), mostly available in the *coloc-stats* interface (4). Those methods are usually based on shuffles or on a statistical model. Challenges in such approaches have been summarized in a recent review (5). Recently, we proposed another type of method involving Monte Carlo fitting of a Negative Binomial distribution while keeping inter-region distances, proven to be more resolutive than previous approaches (6). However, considering only pairwise overlaps will not reveal higher order associations, that is to say associations between a query interval set and multiple reference sets simultaneously. Indeed, most chromatin components such as transcriptional regulators or histones are known to work in combinations and form complexes (7) when binding to the genome. As such, a method is required to rigorously evaluate those combinations. Pairwise overlaps are sometimes used to build association networks (8) but this can be misleading, as an association of a regulator A with B and of B with C does not necessarily mean A and C will be found in the same complex in real conditions.

However, the problem of the significativity of multiple overlaps is rarely tackled. Some existing approaches include *MULTOVL* (9) which uses empirical *P*-values determined from shuffling the region sets to determine the statistical enrichment of higher-order associations. Furthermore, simply evaluating the enrichment of all *n*-wise combinations of *k* sets returns up to 2^*k*^ possibilities, which can be hard to parse. To filter those, other current approaches such as *TFCoop* (10) look for combinations of factors that best explain a given factor, but uses linear regressions which does not show the diversity of existing complexes, instead giving a weight to each set.

Itemset mining, which groups many methods aimed at identifying patterns between sets (11), i.e. when an element of set A is present, sets B and C are often present as well) has also been used to identify interesting combinations of genomic regions (12). For instance, *GINOM* selects *n*-wise itemsets that best explain the query region set (13). A more distant parallel can also be drawn to *ChromHMM* (14), which however divides the genome in mutually exclusive states without hierarchizing combinations. Although itemset mining is mostly performed with tree-based algorithms (15) such as *apriori* (16), some advances are made with non-negative matrix factorization, including inferring TF (transcription factors) combinations (17), and with dictionary learning (18).

Another reason to use itemset mining to identify combinations of interest is the presence of noise. For example in ChIP-seq, which is a technique used to locate binding sites of proteins on the genome, there are known difficulties resulting in false positive peaks, either for biological or technical reasons (19). This may complicate analysis leading to spurious results. Some methods seek to correct the noise, sometimes also leveraging combinations between sets (20). In particular, matrix factorization methods are quite effective although costful on such noisy data (21).

However, using itemset mining to find combinations of interest based on a criterion and assessing their enrichment are two different approaches, which are worthwhile to now be combined. This paper proposes a method named **OLOGRAM-MODL** to leverage both, by calculating the significance of mined combinations of overlaps of interest. OLOGRAM is a statistical framework that evaluates the statistical enrichment of a given combination of sets overlapping, while MODL is an itemset mining algorithm used to select the most representative combinations. We also showcase the approach both on artificial and biological data through several experiments.

## MATERIALS AND METHODS

As an extension of OLOGRAM (6), OLOGRAM-MODL (*OverLap Of Genomic Regions Analysis using Monte Carlo - Multiple Overlap combinations with Dictionary Learning*) can now process overlaps between *n* ≥ 2 sets of genomic regions. We begin by introducing some notations and definitions:

Definition 1 (Set of genomic regions). Let *A*_*i*_ be a genomic region, that is a position interval on the genome (e.g. *A*_*i*_[100_1_; 200_1_] = ‘chromosome 1, base pairs 100 to 200’). Then, the set *A* = {*A*_1_, *A*_2_, ...} is defined as a finite set of individual genomic regions.

Definition 2 (Operator of intersection +). In this paper, the operator + over sets of genomic regions, e.g. *A* + *B*, is used as a convenient notation to designate the intersection of regions from sets A and B (see example below). It does not correspond to the Minkowski sum.

Definition 3 (Combination). A combination γ = {*A* + *B* + *C*} is defined (is non empty) whenever genomic regions included in *A*, *B* and *C*, embed at least one common genomic position. Combinations can be defined on any *n* ≥ 2 sets.

For example, let *A* = {*A*_1_[100_1_: 200_1_], *A*_2_[500_1_: 550_1_]} and *B* = {*B*_1_[480_1_: 520_1_], *B*_2_[100_2_: 210_2_]}. Now consider the combination γ_1_ = {*A* + *B*}: it combination is defined over {*A* + *B*} = {Δ_1_[500_1_: 520_1_]}. The intersection set contains only one contiguous region of 20 base pairs, therefore *S*(γ_1_) = 20 and *N*(γ_1_) = 1

Definition 4 (Measures over a combination: *S* and *N*). For a given combination γ, we define two measures: *S*(γ) is the total number of base pairs on which this combination is observed, and *N*(γ) is the number of windows (defined as a set of contiguous base pairs) on which this combination is observed.

For each combination γ, the objective is to determine whether it is observed in the real data at a higher frequency than it would be under the null hypothesis (*H*_0_). In this approach, (*H*_0_) is that the positions of the regions in a given set are independent of the positions of the regions in any other set. Within a set, regions are not assumed to be uniformly distributed, since the distribution of inter-region lengths is kept (see later).

**Figure 1.**
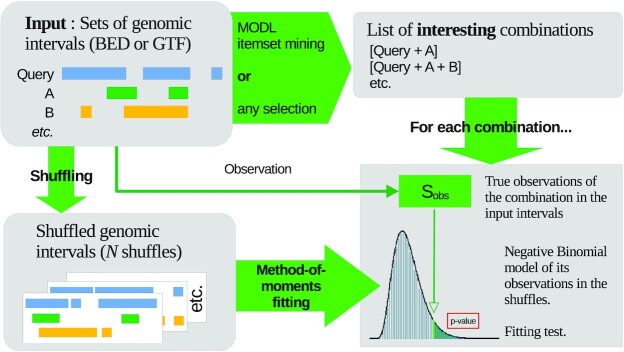
OLOGRAM-MODL pipeline. General pipeline of the OLOGRAM-MODL approach. We fit a Negative Binomial distribution on the number of base pairs on which each combination of elements is observed (see section OLOGRAM enrichment analysis). The list of combinations for which this fitting is done can be obtained in different ways. It can consist of all observed combinations, a selection made by the proposed MODL itemset mining algorithm (see MODL itemset mining algorithm section), a selection based on the most frequent or most significant, and finally a custom selection given by the user.

**Figure 2.**
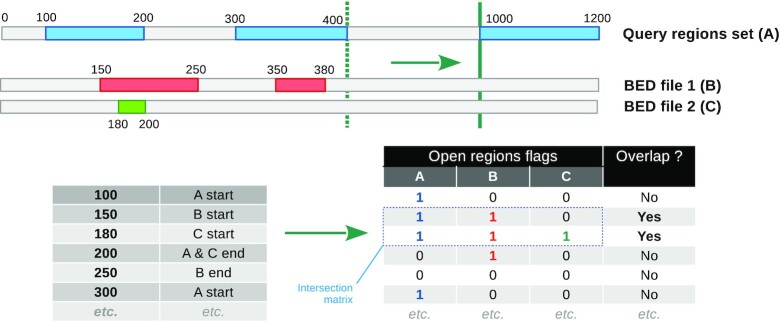
Multiple overlap algorithm implemented in OLOGRAM-MODL. The algorithm belongs to the sweep line family, and takes as input *n* ≥ 2 sets from BED files. It registers critical points (beginnings and ends of genomic regions) and remembers how many such points of each type have been previously encountered. For each observed overlap, the algorithm returns a vector giving the number of regions from each set that are open at this position. The output is the intersection matrix *X*, highlighted in a blue dotted box. It consists of all such vectors *x* such that ∑*x* ≥ 2, meaning at least two sets are open. *X* has one row per intersection and one column per region set.

OLOGRAM-MODL can compute statistically relevant *P*-values for each combination’s *S*(γ) (also for its *N*(γ), but *S*(γ) is generally more relevant). The optional *MODL* algorithm is proposed to filter the list of considered combinations. The general pipeline of the approach is presented in Figure 1. First, the real overlaps are computed and stored in a relevant matrix (Figure 2) from which candidate combinations can, optionally, be easily extracted. Then overlaps are also computed on shuffles (Computing the combination enrichment section) and used to statistically model the enrichment of the combinations (Statistical model discussion section).

### OLOGRAM enrichment analysis

Definition 5 (Enrichment of a combination γ). Let γ be a combination as defined above (Definition 3). Its enrichment, also called fold change, is:

$$\begin{equation*} m(\gamma )= \log _2\left(\frac{S_{obs}(\gamma )}{S_{exp}(\gamma )}\right) \end{equation*}$$

where *S*_*obs*_(γ) is the *S* statistic (nb. of base pairs on which the combination is observed) for this combination in the real data, and *S*_*exp*_(γ) is the expected value of *S*(γ) under (*H*_0_), estimated through shuffling (see below).

As such, the higher the *S*_*obs*_ compared to *S*_*exp*_, the higher the enrichment. Analytic calculation of the enrichment is difficult, since the sets have varying numbers of regions which themselves have varying sizes. Instead, we use a Monte Carlo approach: OLOGRAM’s original principle (6) is to determine the statistical significance of the overlap between two region sets by shuffling them independently many times. This shuffle is done by permutation of the series of regions lengths, but also inter-region lengths. It is possible (and usually recommended) for the user to restrict the shuffling and analysis to certain regions of the genome, which are of interest. OLOGRAM does this by creating a subgenome through concatenation of the interesting regions. If the shuffling is restricted to a sub-genome, regions outside of it are discarded. It essentially amounts to a remapping on shorter chromosomes. Of course, since the shuffling is done only inside the sub-genome, under the null hypothesis we not only assume that the features are independent but also that they can only be located in the sub-genome. It is also possible to lock the positions of the regions in given sets during the shuffling through an explicit instruction in the command line.

The key contribution was using these shuffles to estimate the parameters of an underlying Negative Binomial model, instead of using empirical *P*-values. As such, OLOGRAM has already been shown to be much more resolutive in terms of *P*-value compared to state-of-the-art tools. Here, OLOGRAM-MODL generalizes this principle to overlaps between potentially *more than two* ($n \in {\rm I\!N}$) sets of genomic regions.

To compute the overlaps in both real and shuffled data, we designed and use an algorithm based on the sweep line principle (22) presented in Figure 2. It takes as inputs many sets of regions and returns a matrix representation of their overlaps. Its time complexity is *O*(*N*log *N*), where *N* is the total number of regions in all sets, at initialization, as it requires sorting the critical points through a merge sort (23). However, querying overlaps has a complexity of *O*(∑*n*_*i*_) where *n*_*i*_ is the number of regions in the *i*^th^ set. This is in contrast with interval trees (used in *BEDTOOLS fisher*) whose complexity is *O*(log *n*) per queried region, simplifying to *O*(*n*_2_log *n*_1_) for only two sets.

#### Computing the combination enrichment

For any combination γ, we fit a Negative Binomial model on each measure *N*(γ) and *S*(γ) (Definition 4), and give the *P*-value for the actual observed occurrences to happen by chance.

By default, the approach computes the enrichment of all combinations observed in the real data. To perform a selection among those instead, the MODL algorithm (see MODL itemset mining algorithm section) is proposed. The user can also provide a custom selection, as the intersection matrix itself can be accessed in our Python API to help make that selection. Only combinations containing the specified query region are considered. The counting of each *S*(γ) is further dependent on the transitivity of the partial order defined on combinations:

Definition 6 (Partial order ⪯ over combinations). A combination γ_2_ may include all the sets of a combination γ_1_, plus some others: γ_1_ is the parent and γ_2_ is the child of the relationship, denoted by γ_2_⪯γ_1_. This order induces a lattice structure of combinations where inheritance is defined.

For example, {*A* + *B*} is a parent of {*A* + *B* + *C*} and of {*A* + *B* + *D*}, but not of {*B* + *C*}. Counting a combination γ simply refers to computing its measures *N*(γ) or/and *S*(γ). This can be done on any ensemble of intervals: for the real data, for a shuffle, or anything else. By default, counting a combination is not exact, which means that it includes the observation of its children by transitivity.

Definition 7 (Exact and inexact counting). The exact (single) counting of *S* and *N* of a combination γ does not consider neither its parents nor its children.

Conversely, inexact (transitive) counting of a combination γ takes into account the counting of γ, added with the unduplicated observation of any child of γ at any given genomic position.

Let *A* and *B* be the toy sets of genomic regions given in the previous example where *A* = {*A*_1_[100_1_: 200_1_], *A*_2_[500_1_: 550_1_]} and *B* = {*B*_1_[480_1_: 520_1_], *B*_2_[100_2_: 210_2_]}, and γ_1_ = {*A* + *B*}. In this case, recall that we would have γ_1_ defined over {*A* + *B*} = {Δ_1_[500_1_: 520_1_]}. Now let *C* = {*C*_1_[510_1_: 520_2_]} and let γ_2_ = *A* + *B* + *C*. We have γ_2_⪯γ_1_. As a result of the introduction of the new set *C*, the regions focused by the combinations γ_1_ and γ_2_ have changed. Instead, we now have the following: {*A* + *B*} = {Δ_1_[500_1_: 510_1_]} and {*A* + *B* + *C*} = {Δ_2_[510_1_: 520_1_]}.

An exact counting of γ_1_ will only count on Δ_1_, resulting in *S*(γ_1_) = 10 base pairs. But in a transitive counting (done by default) the regions on which any children of a combination is defined are still counted. Since γ_2_⪯γ_1_, the regions where γ_2_ is defined are counted too, and we now have *S*(γ_1_) = 20 (*N*(γ_1_) = 1 in both cases, since contiguous regions are merged *a posteriori*).

In conclusion, in a transitive counting *A* + *B* actually represents all combinations of type *A* + *B* + Δ (its children), where Δ is any combination of sets of regions excluding *A* and *B*, and is labeled as such. Counting is transitive by default, as this allows easier study of cases where multiple regulators can have a combined effect. An exact counting will instead highlight cases where, for example, the only true biological complex is made of the tripartite association of *A* + *B* + *C*, and *A* + *B* by themselves are not enriched.

#### Statistical model discussion

Consider the regions sets *A*, *B*, *C* of a combination *A* + *B* + *C*. Under (*H*_0_) of no association between the sets, consider the Bernoulli random variables (r.v.) $I_{A_i,B_j,C_k} = \mathbb {1}_{A_i \cap B_j \cap C_k \ne \varnothing }$.

Proposition 1. For any combination γ, if (*H*_0_) is true then a Negative Binomial distribution is a good approximation for the distribution of *S*(γ).

To justify this, we propose the following heuristic proof:

*Heuristic argument* Consider the regions sets *A*, *B*, *C* of a combination *A* + *B* + *C*. Under (*H*_0_), consider the Bernoulli r.v. $I_{A_i,B_j,C_k} = \mathbb {1}_{A_i \cap B_j \cap C_k \ne \varnothing }$. They can be expressed as a product of pairwise $I_{A_i,B_j} * I_{A_i,C_k} * I_{B_j,C_j}$. Those are dependant Bernoulli r.v. for two reasons. First, the locations of the regions are permuted in the shuffles, so if *A*_*i*_ and *B*_*j*_ overlap in a shuffle the likelihood of *A*_*i*_ also overlapping with a different region *B*_*k*_ of the set *B* is greatly reduced, since the regions are merged. Second, if *A*_*i*_ overlaps *B*_*j*_ and *B*_*j*_ overlaps *C*_*k*_, it is likely that *A*_*i*_ also overlaps *C*_*k*_. Let us express this in terms of conditional probabilities: $P(I_{A_i,B_j,C_k}) = P(I_{A_i,B_j} = 1) * P( I_{A_i,C_k} = 1 | I_{A_i,B_j} = 1 ) * P(I_{B_j,C_j} = 1 | I_{A_i,B_j} = I_{A_i,C_k} = 1)$. If one approximates each term as the result of another Bernoulli variable of unknown but fixed probability *P*, one can approximate their products $I_{A_i,B_j,C_k}$ themselves as dependant Bernoulli r.v. of unknown *P*. While calculating the *P* themselves requires the expression of the correlations between the variables, they can be instead estimated via a Monte Carlo approach. Indeed, (6) shows that the sum of dependent Bernoulli r.v. follows a Negative Binomial distribution, which is also true for *S*(γ) = ∑I*Λ_*I*_ where Λ_*I*_ is the length of each intersection.

However, this is true only if the following two conditions are met. First, that Λ follows a log-normal distribution; this is often the case empirically since the log-normal distribution is versatile. Second, that the various *I* of the Bernoulli sum be exchangeable. The pairwise $I_{A_i,B_j}$ can be considered exchangeable because: if *A*_1_ overlaps *B*_1_, it is unlikely to also overlap *B*_2_ and vice versa. But it does not matter which of the two (*B*_1_ or *B*_2_) is overlapping *A*_1_: when taking the joint distribution of all $I_{A_1,B_j}$ over *A*_1_ and all regions in *B*, the probability is the same. The same logical argument applies to *n*-wise intersections by taking the joint distribution over many sets. And in any case, if the pairwise *I* are exchangeable, since a sum of *n*-wise *I* is the sum of their constituent pairwise *I*, it does not really matter.

In practice, this is only an approximation since, for instance, the various regions of *B* will not have the same lengths, nor inter-region lengths. The shuffling contributes to making the *I* approximation work by shuffling the inter-regions lengths. But this still requires that the sets be composed of many regions (to have many inter-region lengths), and that the region lengths remain close to each other, and small compared to the inter-region lengths.

Considering these assumptions, *N* and *S* follow a Beta-Binomial distribution under *H*_0_. In practice it is preferable to approximate it with a Negative Binomial distribution, for both precision and computational complexity reasons that are detailed in the Results and Discussion. This approximation is only asymptotically correct when β and *n* tend to infinity (24) but works well in practice.

In conclusion, we propose that *S*(γ) can be approximated reasonably well by a Negative Binomial distribution of unknown parameters in most application cases, under the above assumptions. This is in large parts thanks to the resampling procedure. Furthermore, the algorithm performs a goodness of fit test (after shuffling) for the Negative Binomials for each combination, in order to exclude the case that the null hypothesis is false because the empirical distribution is not a good approximation of the Negative Binomial. This test is based on Cramer’s V-score (6). This is expanded in Artificial data and comparison to existing approaches section, where we also present examples of empirical distributions being fitted with the Negative Binomial law.

When running the algorithm, the parameters of the Neg. Binom. distributions are estimated based on the empirical mean and variance observed in the shuffles. This is known as method-of-moments fitting. In most cases, 100–200 samples (here, shuffles) are enough to fit a Negative Binomial distribution reasonably well (6), and Supplementary Figure S7.

Poor fits are mainly observed when the combination in question is too rare in the shuffles (when one of its sets covers a too small proportion of the genome), when there are too few regions in a set, or conversely when the shuffling was restricted to a too small genome. Estimated *P*-values will tend to be too conservative in those cases, but fold changes are accurate.

Using a statistical model instead of empirical *P*-values is crucial for combinations containing a large number of sets (i.e. high order), for which the likelihood of observing high values of *S*(γ) in the shuffles will be low. *This also means that one should be careful when comparing enrichment between combinations of different orders* (the order of a combination is the number of region sets it concerns). We expand upon this in the Discussion section.

#### Time scaling

The scaling of OLOGRAM (just OLOGRAM, the scaling of MODL itself is discussed in the next section) is multifaceted. It consists of three main steps: (i) shuffling the sets and computing their intersections (Computing the combination enrichment section), (ii) computing for each combination of interest whether it is transitive with all other combinations in the shuffles and (iii) fitting the Negative Binomials.

Runtime depends on *e*_*i*_, the expected number of intersections to be computed in each shuffle, which in turn depends on the density of each source region set. If we also have *e*_*n*_ the number of different combinations encountered in the shuffles and *e*_*t*_ the number of different combinations in the true data, globally step (i) scales in *O*(*e*_*i*_) on top of the already advantageous scaling of the sweep line algorithm, step (ii) scales in *O*(*e*_n_*e_*t*_) and step (iii) in *O*(*e*_t_*e_*i*_). Steps (ii) and (iii) are generally only time consuming if *k* (number of sets) is large.

### MODL itemset mining algorithm

Up to 2^*k*^ combinations are possible with *k* sets of regions, (although usually much fewer are observed in practice). Furthermore, simply sorting by *P*-value may not be helpful, since the most enriched combinations are not necessarily the most interesting: a combination that was expected across 2 base pairs but observed on 2000 bp will be very enriched but may still be one of the rarest observed combinations and not be biologically very relevant. Both these facts can make interpretation of the results difficult.

To alleviate this, by default combinations are sorted by their observed *S*(γ) (number of base pairs) in the true data. To go one step further, we introduce the optional MODL *(Multiple Overlap Dictionary Learning)* algorithm for itemset mining (Algorithm 1). In OLOGRAM-MODL, it can be used to pre-select combinations of sets which are of interest, hence reducing afterward the total number of enrichment computations and making the results easier to interpret.

Algorithm 1 Multiple Overlap Dictionary Learning (*MODL*) algorithm for combination mining 
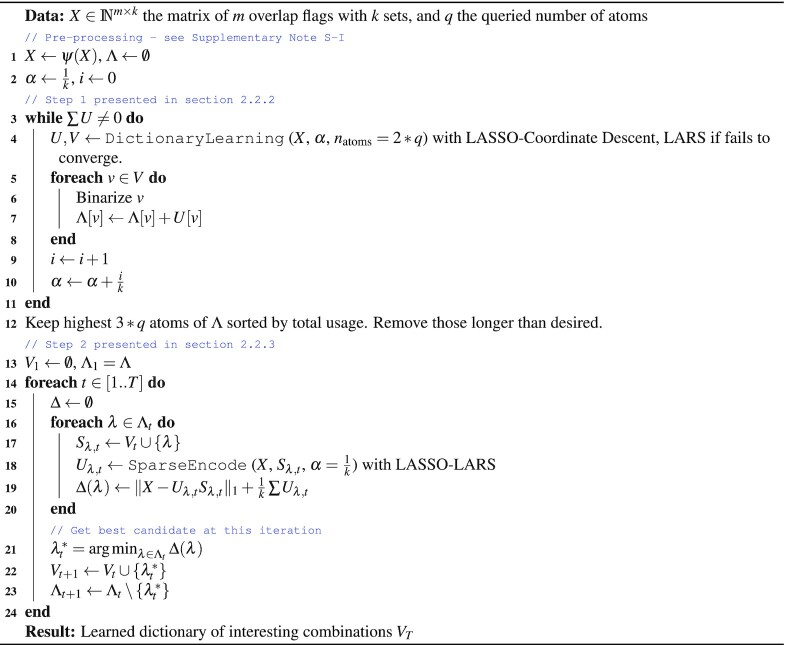


#### Extraction of relevant sub-complexes with dictionary learning

In the OLOGRAM-MODL approach, the input matrix of MODL is the matrix of overlap flags provided by the algorithm in Figure 2. It has one row per overlap and one column per set in the real, non-shuffled data. However, any matrix matching this format can be used.

**Figure 3.**
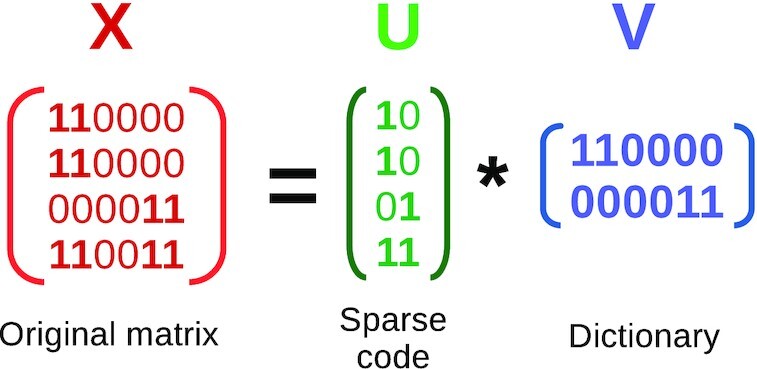
Principle of itemset mining via dictionary learning. Dictionary learning is a special case of matrix factorization, where the goal is to learn the sparse code *U* and the dictionary *V* from the data *X* under certain constraints, minimizing the reconstruction error. The dictionary should have a small number of rows (atoms). It can be clearly seen how the atoms (rows) of the learned dictionary *V* can be mined for frequent itemsets in the data, giving sets that are often present together. Although this figure pictures binary matrix, any real-valued matrix *X* and *U* can be used. Note that in real dictionary learning, the rows of *V*^2^ sum to 1, but the principle is the same as illustrated here. In OLOGRAM-MODL, *X* corresponds to the matrix of intersections as created in Figure 2, taking only all rows where overlap is ‘Yes’. Each row of the dictionary *V* is a potentially relevant combination γ.

Figure 3 shows the principle of dictionary learning (21) as used by MODL, which is a factorization matrix problem with sparsity that entails solving:

$$\begin{eqnarray*} (U^*,V^*) = \mathrm{arg\, min}_{U,V} \frac{1}{2} \Vert X-UV\Vert ^2_2 + \alpha \Vert U\Vert _1 \\ \text{subject to } \Vert V_i\Vert _2 = 1 \text{ for all } 0 \le i \le n_{\rm{atoms}} \end{eqnarray*}$$

It shows that relevant itemsets can be extracted from the atoms of the dictionary *V*. A dictionary is composed of atoms (its rows), which are used to rebuild richer words (rows of *X* are observed combinations). Here, atoms represent biologically relevant sub-complexes. Adding redundant atoms (i.e. (11) if (01) and (10) are already present) if they improve the rebuilding can be warranted to represent the entire complex.

MODL is less vulnerable (but not immune) to noise than usual tree-based approaches, since it is based on matrix factorizations (25), (26) and Results and comparison section. Noise is defined broadly as any factor that causes an observation different of the biological reality. It can be fully random, but since matrix factorization focuses on strong signals it can still accommodate a coherent signal acting as noise, as long as it is weak compared to the main biological signal. Biologically, this can be due to ChIP-seq false positives, or any number of causes depending on the experiment. As such, the learned atoms can be buildings blocks referring to parts of a complex (group of sets), like in the third line of Figure 3 instead of minor variations of the combinations.

Definition 8. The usage of an atom *V*_*j*_ in the rebuilding of a given word $\hat{X}_i$ (where $\hat{X}_i = U_iV$) is given by *U*_*i*, *j*_, and is simply the multiplicative coefficient associated to it in this particular rebuilding. An atom’s total usage is thus equal to $\sum _i^m U_{i,j}$.

However, the atoms present in the learned dictionary heavily depend on the parameters, mainly α. As such, MODL consists of two steps. In step 1, MODL performs various reconstructions with dictionary learning with various sparsity constraints to produce a library of candidate atoms. In step 2, a greedy algorithm builds the final selection by getting the best encoding candidates using the maximization of a local function with regularization. In the following, let *k* be the number of sets in the matrix $X \in {\rm I\!R}^{m \times k}$ of *m* observations, and *q* the queried final number of itemsets is a parameter of MODL.

#### Library creation through sparse dictionary learning

Since MODL’s goal is to best reconstruct the input matrix, its cost scales with matrix size, and it emphasizes combinations found in the most frequent observations. To mitigate this, a compressed version of the input matrix *X* is processed instead, called a smothered matrix, where the abundance of each combination is quadratically reduced. The full pre-processing applied to *X* is detailed in Supplementary Note S-I. This results in a pre-processed matrix called ψ(*X*).

Then, the first step of MODL itself is to compute a library of candidate atoms. This is done by performing several successive factorizations on ψ(*X*) as explained in Extraction of relevant sub-complexes with dictionary learning section. The reconstructions are repeated with different sparsity constraints α to get candidate atoms of various lengths. At each iteration, α is increased by *i*/*k* where *i* is the iteration number: this resutls in the learning of longer atoms because a higher α allows less atoms to be used. This step stops once α is so high that the total usage of all atoms is zero. More details about this step are presented in Supplementary Note S-11.

#### Greedy algorithm for combination selection

Now, the final *q* combinations constituting the final dictionary *V*_*T*_ will be selected by iteratively adding the atoms maximizing the fidelity *f* of the rebuilding:

$$\begin{equation*} V_T = \mathrm{arg\, max}_{S} f(S) \text{ , where } f(S)= -\Vert X-US\Vert _1 + \alpha \sum U \end{equation*}$$

At each iteration, the best atom λ* of the library Λ is greedily added to the dictionary *V*_*t*_, which is initially empty. For that purpose, at each step *t*, a two-stages optimization process is performed which first computes all the sparse approximations for all remaining candidates *U*_λ, *t*_ of *X* using the current dictionary *S* = *V*_*t*_∪{λ}, and which then chooses the λ* that minimizes the difference *d*_1_ between *X* and its approximation *U*_λ, *t*_*S*_*t*_, where:

$$\begin{equation*} d_1(X,\tilde{X}) = \Vert X - \tilde{X} \Vert _1 + \alpha \sum U \end{equation*}$$

Unlike in step 1 where U*, V* were optimized conjointly, here the sparse coder will find U* for a given *V*_*t*_. More details are presented in Supplementary Note S-III. We also discuss in this note how the problem of finding S* = arg max_*S*_*f*(*S*) admits a good submodular approximation and provide a proof sketch for this. This means that the greedy algorithm described above provides a good solution.

### Implementation and availability of the method

As an update of *OLOGRAM*, the code is written in Python 3, with some performance-critical tasks in Cython and C++. To preserve RAM when working with large files, the shuffles are divided into batches. However, OLOGRAM must still remember all computed intersections within a run; hence, we also permit merging different runs as superbatches if necessary, although fitting is not assessed then. The demonstration examples take a timescale of minutes to run, up to hours for very large cases (see Running times section) on a 4-core 3.5 Ghz processor.

For the MODL subroutines of dictionary learning and sparse coding, the *Scikit-Learn* implementation is used (27). OLOGRAM-MODL is accessible through the command line interface of *pygtftk* (28) which is available on Bioconda. The GitHub also contains documentation with more information. The integration with the *pygtftk* suite of tools allows easy use in bioinformatics pipelines and easier extension. A detailed list of its source code files and their roles is presented in Supplementary Table S5.

The tool will output one set of statistics per combination of sets of interest in a TSV (Tab Separated Values) format that can be manually edited. Then, an *ologram_modl_treeify* plugin creates visual representations of the results of a multiple overlap analysis, used to generate Figure 5. The MODL algorithm can also be used as a standalone combination mining algorithm through the API.

### Data and results

We use two main types of data to produce the results of this study. Two different types are used: either a set of genomic intervals as BED files, or a representation of the overlaps between those sets of intervals as a matrix *X* where each row is a combination. The details about the creation and the detailed sources of this data is presented in Supplementary Note S-IV.

Artificial datasets are generated with a known ground truth, so we can use them to demonstrate and quantify the behavior of our algorithms. Those are used in the results presented in section Artificial data and comparison to existing approaches section. For the artificial matrices, they have a large number of rows to perform a large scale experiment and ensure the results are not due to random chance, and the itemsets selected represent biologically interesting cases, with a diversity of complexes and two overlapping ones.

Conversely, we also use three biological datasets to produce the results of Biological results section: human and murine transcription factor binding sites ChIP-Seq data, as well as human sc-ATAC-seq data. The two first datasets serve to demonstrate OLOGRAM’s utility in discovering complexes of regulator, and the third demonstrates how it can scale to larger problems. When selecting the transcriptional regulators to study, we tried to avoid cherry picking by using a wide variety of TRs, and including many commonly studied ones. We present the full list in Supplementary Data.

The complete workflow used to produce the Results of this study, along with the full data used, is available as a Snakefile (Snakemake file, (29)) at https://github.com/qferre/ologram-modl_supp_mat. Users can re-use and adapt the commands presented there for their own workflow.

## RESULTS

In summary, OLOGRAM-MODL takes an input a set of genomic regions (usually in BED format) and will determine for each encountered combination of genomic regions whether it is enriched, meaning whether it is encountered more than expected by chance. The MODL algorithm can be used to restrict the combinations for which this is calculated to combinations of interest (custom selections are also possible).

The first goal of the experiments presented in this section is to validate our statistical model and our itemset mining algorithm. We do so using artificial data for which the ground truth is known, and the results can be compared to. We also compare to existing approaches. In the second part, we show that OLOGRAM-MODL is not only able to deal with true biological data but also a fair way to discover relevant complex of genomics regions and get novel insights.

### Artificial data and comparison to existing approaches

First, we want to establish that our statistical model is valid in the context of combination enrichment. We also want to show that the MODL algorithm can find relevant itemsets in situations representative of real use cases. To do so, we benchmark OLOGRAM-MODL against data with a known ground truth to which the results can be compared. The datasets used will refer to the data defined in Supplementary Note S-IV, see this note for more details.

A comparison between the functionalities of OLOGRAM-MODL and of existing available tools is presented in Supplementary Table S1 and in the rest of this section. We show that OLOGRAM-MODL is easy-to-use and brings novel insights compared to existing approaches.

#### OLOGRAM statistical model

We use the BED files representing artificial regions, with an inexact counting. In the regular artificial data, OLOGRAM correctly identifies the associations between sets: as a general rule, sets that have strong overlap with each other are seen as enriched, and vice versa. Notably, the query was found enriched with its subsets but not with the negative control. Detailed results are presented in Supplementary Figure S1.

##### Precision

For the studied *n*-wise combinations, the *S* statistic (number of overlapping base pairs) also indeed follows a Negative Binomial distribution, which confirms the assumptions of the statistical model. The findings of the previous paragraph also apply. Full histograms are presented in Supplementary Figure S2. The precision of the *P*-value estimated through this depends on the quality of the fitting. This in turn depends on the number of shuffles, but 100–200 shuffles are usually enough for a good approximation (6). To that end, the goodness-of-fit is systematically assessed for each combination.

The precision of the fitting for the tails of the distribution is presented in Supplementary Figure S7. For pairwise, the empirical distribution is visibly close to a Negative Binomial. The precision is very good for high values of *P* up to *P* = 10^−5^ which is far below usual significance thresholds, and only drops on the far end of the tails of the distribution. But the underlying distribution has the general shape of a Beta distribution (see the C subfigure), meaning the estimated *P*-values will still be proportional to the real underlying ones.

Furthermore, our Negative Binomial model is less accurate when there is a small number of regions in the sets (sufficient quality with roughly 1–2 thousand regions per chromosome), and that it is valid only asymptotically. Nevertheless, the Negative Binomial model is still preferable, for reasons that are expanded upon in the Discussion section. If there are few expected overlaps, the distribution can be skewed to the left (as in case C of Supplementary Figure S2) and be more difficult to fit since there are few nonzero observations, but the underlying distribution is nevertheless reasonably well approximated by a Negative Binomial.

##### Comparison

Compared to MULTOVL, we show in Supplementary Table S2 that there is only a slight difference in our shuffling statistics, owing to a different shuffling model. Negative Binomial distributions with the same parameters closely match MULTOVL’s empirical *P*-values. On cases with a high *P*-value (*P* > 0.05), meaning only a low resolution is needed, our Negative Binomial model is validated. On harder cases MULTOVL’s precision plateaus at 1/*n*_*s*_ where *n*_*s*_ is the number of shuffles, while OLOGRAM is more resolutive. Furthermore, MULTOVL gives statistics by multiplicity (not which sets are open at a given position, only how many) and not for each combination as OLOGRAM does.

##### Running times

A full run of 1000 shuffles in the large ‘coarse’ artificial dataset took 40 s on a laptop (i7-7820HQ processor, single thread), against 10 s for MULTOVL. Typical runtimes are in the order of a few minutes, such as for the example in Regulatory complexes in murine promoters section. In extreme cases, such as the very large data use case of Overlap between single-cell ATAC-seq in PBMC data section with 75+ large files, the runtime was 1.5 h per 4 shuffles, mostly due to the analysis in steps (ii) and (iii) (as defined in Time scaling section) and not the shuffles themselves. In general our running times are of similar orders of magnitude compared to existing tools. For all these cases, detailed runtimes, data characteristics and computer specifications are presented in the legends of the Supplementary Figures.

#### MODL

OLOGRAM will quantify the enrichment of any combination encountered, or of a custom selection. But with *k* sets, there are up to 2^*k*^ possible combinations, although in practice far fewer are encountered. To help select among those, we propose an ancilliary itemset mining algorithm called MODL. In this section, we show that MODL can help select relevant combinations.

##### Results and comparison

We compare MODL to other itemset mining algorithms: *apriori* and *FP-growth* are exhaustive algorithms that return all the correct itemsets, and most improvements today are focused on runtime (11). We also compare to LCM (closed itemset miner) and CL-MAX (approximate itemset miner). The algorithms’ usefulness in identifying the underlying combinations used when generating the artificial overlap matrices (see Data and results section) is compared. We compare the status of each combination in MODL (selected or not, and candidate rank) to the rankings and selections given by the other algorithms. Even when applying 12% uniform noise, MODL still returns the correct combinations.

Results are presented in Supplementary Table S3. The top three combinations found by MODL are indeed the three complexes defined when generating the data: *AB*, *ABCD* and *EF*. Other algorithms give them a lower ranking, because if the rule *ABC* is true the rules *AB* and *ABC* are equally true.

Itemset miners in general are vulnerable to noise. Approximate itemset miners (such MODL purports to be) are proposed as a solution (25). Indeed, when using a clustering-based approximate itemset miner (CL-MAX, (30)), results that are close to MODL’s. However, CL-MAX is more vulnerable to a poor choice of hyperparameters.

We also compare OLOGRAM with GINOM on their demonstration data. There is a link between the fold changes in OLOGRAM and in GINOM, as shown in Supplementary Table S4. The combinations selected by the two approaches differ somewhat since MODL is not based on enrichment, but MODL’s selection is still relevant, and there are commonalities between the two. MODL behaves as would be expected of a frequent itemset mining algorithm, in that it seeks to rebuild the original data matrix and does not select sets alone but with their co-localized sets (i.e. not selecting set 5 alone).

To perform a relevant comparison, we also ran a supervised version of MODL to perform variable selection for a Naive Bayes classifier predicting the presence of the query regions. Variable selection in Naive Bayes is known to be submodular (31), and as such this constitutes a good use case of MODL. Results are closer to GINOM (stronger emphasis on sets 4 and 5). This is subject to refinement and parameter selection but serves as a proof-of-concept for a supervised MODL (the API for this is explained in the documentation).

MODL, when unsupervised, has a bias towards the most abundant combinations in the data, instead of those with highest support. As it is designed to mine for complexes, it also tends to return too broad potential correlation groups (cf. Library creation through sparse dictionary learning section) or simply noise patterns. This loss of granularity is a known necessary drawback of Approximate Itemset Mining approaches (25), but the normalization of the atoms by their squared sum helps correct this problem (cf. Greedy algorithm for combination selection section), and we discuss further ways to correct this in Supplementary Note S-III.

##### Scaling

To produce these results, MODL’s time cost remains reasonable in most use cases, a few minutes at most on a laptop for the typical cases: a few files of tens of thousands of regions. Details of MODL scaling and time cost are presented in Supplementary Figure S3. It can become large due to the large number of factorizations performed: MODL scales in *O*(*k*) with the number of sets and *O*(*q*^2^) in the number of queried combinations (atoms).

For reference, we also compare the elementary operation of MODL (one instance of dictionary learning, DL) with *apriori* and *FP-growth*. Unlike them, dictionary learning scales linearly with the number of sets. DL also scales linearly in the number of queried atoms, while tree-based algorithm scale exponentially with support until they reach a plateau. Run time on DL also increases with the entropy (amount of information) present in the data, as the stopping is convergence-based. Details are presented in Supplementary Figure S4. While DL may be more expensive for low *k* and several thousand intersections (i.e. transactions) that are the typical use cases for OLOGRAM, time costs remain in the order of seconds for all (32).

### Biological results

Having demonstrated that OLOGRAM-MODL’s behavior matches our expectations, we now apply the tool to a variety of biological problems to demonstrate that it can present relevant insights in different use cases, while remaining easy to use. Nevertheless, the rigorous validation of our approach rests on the results obtained with artificial data, where the ground truth is known. This section on biological data should rather be seen as illustrative of the possibilities of the approach when applied to biological problems.

#### Regulatory complexes in murine promoters

We begin with a fundamental use case for OLOGRAM: the analysis of regulatory complexes. We perform a search in murine promoters, by looking for the transcriptional regulators (TRs) among our selected TRs that are associated with three different query TRs: CTCF, NANOG and IRF1. The shuffling is restricted to estimated murine promoters (see Data and results section). MODL is not used here yet. In each case, each query TR is run against all other selected TRs, without cherry-picking. The colors and classification are only used to interpret the results *a posteriori*.

We would expect that CTCF (33) would be involved in combinations with factors found in insulators (RAD21, SMC) as well as other factors due to its plurality of roles. IRF1 (34) should be associated with factors involved in interferon response (IRF9, STAT). Finally, NANOG (35) is expected to be associated to stem cell developmental factors (KLF4, POU5F1). Other associations should nevertheless be present since those TRs are known to have multiple roles. Noise and imprecisions could also introduce spurious associations.

**Figure 4.**
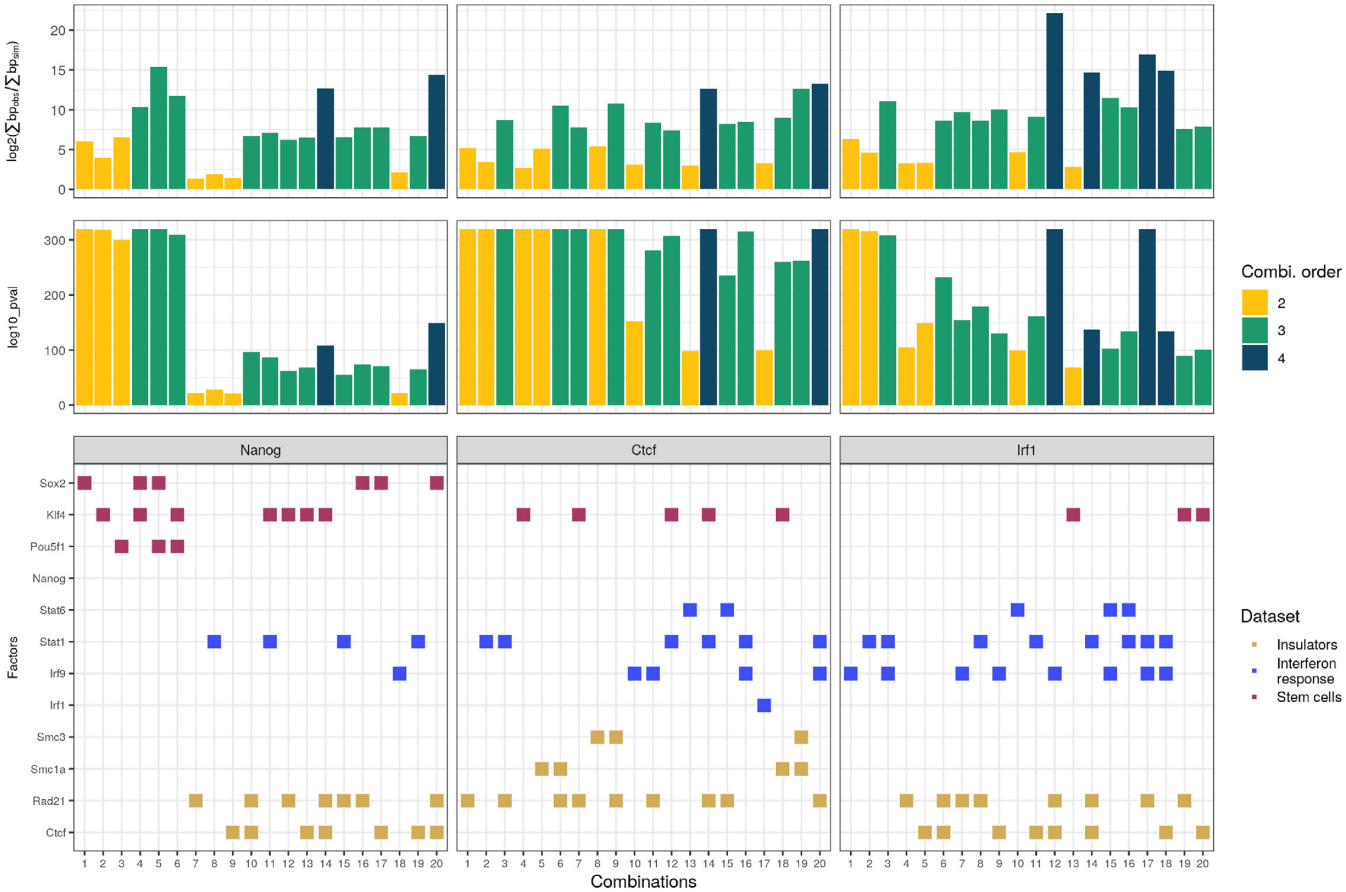
Regulatory complexes in murine promoters. Each histogram bar corresponds to OLOGRAM statistics for one combination of regulators. From top to bottom, the first figure gives its fold change *m*(γ), the second gives the OLOGRAM *P*-value (log_10_), and the third figure details the composition of each combination. We separate the results for the three different queries.The color of the histogram bars represent the order of the combination (its number of sets/TRs). The ranking given in the *X* axis, from 1 to 20, is the ranking of the combination when sorting by true *S*(γ) (number of base pairs on which the combination is observed in the true data), not by *P*-value. In the third figure, each Transcriptional Regulator is colored according to its general role. As expected, we find that NANOG is more associated with regulators involved in stem cells, IRF1 with interferon response ones, and CTCF with insulators.

Results are presented in Figure 4. When ordering the combinations by true *S*(γ), meaning their number of base pairs in the true non-shuffled data, we find the expected complexes first, especially for NANOG. In particular, when comparing combination of the same order (number of sets), those we expected have consistently higher fold changes and more significant *P*-values in OLOGRAM (see Computing the combination enrichment section). This example shows OLOGRAM can be used to robustly identify biologically meaningful combinations.

#### FOXA1 and its regulatory complex in MCF7

In this analysis, we now showcase how the MODL algorithm can help identify new biological complexes. We also show a proposed tree (or more accurately, directed acyclic graph) representation of the combinations, meant to facilitate interpretation.

The combinations of TRs associated with the transcriptional activator FOXA1 are studied here, in the *MCF7* breast cancer cell line. FOXA1 is known to interact with chromatin as a pioneer factor. In many types of breast cancer, such as MCF7, it is also known to act as a pioneer factor to the regulator ERα (ESR1, (36)). Conversely, it is a downstream target of the regulator GATA3 in breast cells (37).

We compare FOXA1 (as the query) against a selection of TRs drawn from the most common in MCF7 as well as some expected TR interaction partners. As the regions considered (TR binding sites) cover a small proportion of the genome, the shuffling is restricted to a subgenome of interest to ensure the longer combinations still have a reasonable chance of occurring under (*H*_0_). This subgenome is made of estimated pseudo-Cis-Regulatory-Modules, defined as the merged regions for all considered TRs.

**Figure 5.**
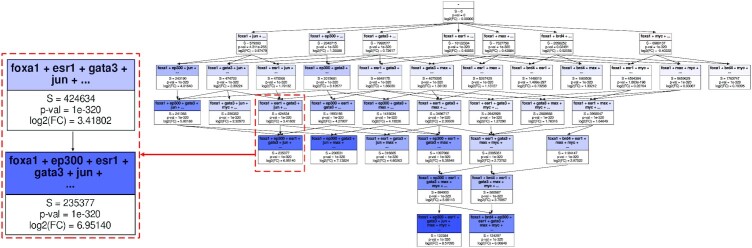
FOXA1 Regulatory tree. Example of the structure of the output graph that pictures combination enrichment for FOXA1 in MCF7, with a zoom on a relevant part. For each combination, the number of base pairs over which it is found in real data (*S*) is presented, followed by the log_2_ of the fold change and corresponding *P*-value according to a Negative Binomial model. The color gradient is based on the fold change. The combinations displayed are a selection based on the selection by MODL with some combinations manually added. This figure is created by our *ologram_modl_treeify* plugin based on an edited TSV result file given by the *ologram* command.

An illustrative selection of combinations is presented in Figure 5. The expected correlators of ESR1 and GATA3 are indeed found enriched. MAX and MYC, known to form a complex (38), are as expected found more enriched together than separately. The graph representation highlights that same-length combinations containing ESR1 and GATA3 but without either EP300 or JUN have lower fold changes. This suggests that they are all an important part of a FOXA1 regulatory complex. The graph also highlights the role of ESR1 through downward closure: for example, the combination FOXA1 + GATA3 + MAX is observed on 4 million base pairs, but 3.5 million of these observations are in fact FOXA1 + ESR1 + GATA3 + MAX.

MODL (with *k* = 8 and *q* = 20) selected relevant shorter combinations first, although they are not the most enriched. Its selection without manual additions is presented Supplementary Figure S5. EP300 and JUN are less prominent in the selection as they are comparatively rarer, but indeed found as part of their regulatory complexes (large proportion of their total *S*). Conversely, the frequent MAX and MYC are more represented. MODL tends to select closed itemsets (for instance, EP300 was not selected alone), but this is admittedly not always true.

In conclusion, this analysis highlights the regulatory complexes formed by the interactions between FOXA1 and others regulators. Rather than saying ESR1 is associated to FOXA1, it would be more correct to say it is part of a regulatory complex to which FOXA1 also belongs. This deserves further study as such associations are, to our knowledge, not explored in the literature. This illustrates how OLOGRAM-MODL can be applied meaningfully to certain biological problems.

#### Overlap between single-cell ATAC-seq in PBMC data

OLOGRAM-MODL is also applicable to more systematic studies. In this section, we demonstrate that OLOGRAM can be applied with a larger number of region sets (*n* > 70). To do so, we consider 75 randomly selected cell samples from single cell ATAC-seq PBMC data. We want to see if the ATAC-seq sites, denoting chromatin accessibility, are similar between cells of the same type. This study was performed by merging different runs in order to reduce the RAM cost at any single time, since we were working with many large files.

We consider the enrichment for each possible combination γ of cells depending on its heterogeneity. For example, in the combination *A* + *B* + *C*, if all its member sets are are sc-ATAC-seq sites from CD4 cells the combination is considered homogeneous. Let *D* and *E* be ATAC-seq sites for respectively a CD14 and pre-B cells. Those cells are very different and have been grouped in different superclusters. As such, the combination *A* + *D* + *E* is called ‘heterogeneous’ since it concerns the overlaps of open genomic sites from very different cells.

We compare combinations one order at a time, and results are presented in Supplementary Figure S6. We find that homogeneous combinations are more enriched. Indeed, it stands to reason that the open genomic sites of cells from very different types (different superclusters) would be less similar than the sites of cells of similar type (same supercluster). However, the difference is relatively small due to high individual variations in the data.

## DISCUSSION

The *OLOGRAM-MODL* approach consists of two steps. We combine an optional itemset mining algorithm to find interesting combinations, with a statistical model to determine the enrichment of the relevant combinations, asserting whether this combination occurs in the real data across more base pairs that would be expected by chance.

The integration with the *pygtftk* toolkit facilitates complex queries in bioinformatic worfklows. As a command line tool whose dependencies can be handled by *conda*, it is convenient to run on clusters with reproducible workflow managers such as Snakemake. Thanks to the use of a Monte Carlo approach and to C++ optimization, OLOGRAM-MODL can even be run on a laptop.

### Statistical model

The precision of the *P*-value given by OLOGRAM depends on the quality of the Negative Binomial fitting, but should be accurate enough in most cases (see OLOGRAM statistical model section). Inaccuracies come mostly from how precisely the variance and mean are estimated, and from how precisely the model approximates the true underlying distribution. As a result, a *P*-value of 1E-200 in OLOGRAM does not mean the *P*-value has been estimated to a precision of 200 significant digits.

The Negative Binomial model is only asymptotically true, but in practice it is a good enough approximation for the underlying Beta Binomial. Above *P* > 0.001, far below usual significance levels, the fitting is good enough that there is no significant difference in the *P*-values, hence small false positive risks. In any case, the order of *P*-values remain unchanged: if a combination 1 has a lower true *P*-value than combination 2, its estimated Neg. Binom. *P*-value will be lower too, since a Negative Binomial is a special case of a Beta Binomial.

Regardless, fitting directly a Beta Binomial is less desirable since its density function calculation relies on computationally expensive numerical approximation, and we cannot fit the ‘*n*’ parameter (theoretical maximum). Nevertheless, OLOGRAM also gives the *P*-value for *S* as fitted through a Beta distribution. The Beta is a good approximation for a Beta Binomial (39) since *n* is usually very large (>10 000). However, Beta distributions are harder to fit, since unavoidable small errors in the estimation of the skewness and kurtosis will propagate (40). With our usual number of shuffles (hundreds) it is very imprecise (see Supplementary Figure S8) and is only preferable with thousands or more of shuffles. Maximum Likelihood Estimates are not used since they are less robust in general, and even a small difference could result in very different *P*-values. In conclusion, although the Negative Binomial model is approximate, we find it to be preferable in practice.

With a large number of region sets, and/or with regions covering a small proportion of the genome (such as Transcriptional Regulator binding sites), longer combinations (and children of enriched combinations) will have small expected overlaps and as such higher enrichment, as seen in Results. Those combinations may not be the most representative configurations taken by the region sets, and one must be very cautious when comparing *P*-values between combinations of different orders (recall that the order of a combination is the number of region sets it concerns). Helping select among those is also part of MODL’s purpose. In any case, this is why we (by default) emphasize frequent combinations in the displayed results by sorting them by their *S*(γ) in the true data.

Necessary elements in regulatory clusters, as well as master regulators (41), are emphasized through a tree-based representation (Results section) and transitive counting. This representation highlights, for each combination, the increase in enrichment brought by adding a given set to the combination. We expect this to be useful in studying Cis-Regulatory Elements as *n*-wise clusters of regulators and moving away from only considering pairwise associations. Furthermore, we also recommend to restrict the shuffling to a smaller sub-genome of interest (as also recommended by (9)), for example only to enhancers or promoters, or to the merged regions of selected candidate set. No more than around 20 sets at once should be considered, keeping in mind that biologically relevant complexes of TFs are usually made of a maximum of 5–6 factors.

Finally, the fitting could be inaccurate for many other unforeseen reasons. Indeed, our model rests upon certain assumptions (i.e. exchangeable variables, sufficient nb. of regions, etc.). The null hypothesis can be rejected if any of these assumptions is not verified, or merely because the approximation holds only asymptomtically. The fitting test is the key: if, when performing the shuffles, it is found that the distribution of *S*(γ) under our shuffling model does not follow a Negative Binomial, it will be said. Then, if the hypothesis is rejected (low *P*-value) but the fitting was good, it can be assumed that the sets are not independent. Admittedly, the fitting test does not deeply fit the tails of the distribution, but it shows if the general shape is close enough. As such, OLOGRAM also gives empirical *P*-values (on the empirical distribution of *S* in the shuffles) if you do wish instead to sample deeply. But as can be seen in Supplementary Figures S2 and S7, the match is usually good. Regardless, a Negative Binomial distribution is a natural choice to model counts of observations with more variance than a simple Poisson distribution. This was an intuitive result at first, that was revealed to be practically applicable and theoretically justifiable.

### Itemset mining

The MODL itemset mining algorithm can be used to focus on elementary combinations of interest. It leverages matrix factorization techniques for their robustness to noise, which is a widespread problem in biological assays. This also means that, compared to usual itemset miners, MODL is focused on learning biological complexes as coherent units, and not simply association rules. This behavior is more relevant for a biological analysis, where identifying only a portion of a regulatory complex would not be as useful. This entails learning compromise combinations (see Supplementary Table S3), as such the queried number of sets should be kept close to the actual expected number.

Note that no matter which combinations are identified by MODL, the enrichment results do not change. MODL’s time cost remains reasonable in most use cases. It also scales better at high *k*, compared to methods such as the stepwise selection of models used in GINOM that may need to explore a parameter space that has 2^*k*^ combinations. It is also more focused on denoising compared to usual itemset miners, although our pre-processing will nevertheless increases the sensibility to noise by emphasizing rarer combinations: its use is a compromise between denoising and not ignoring the rarest combinations.

MODL remains exploratory and a first contribution open for future research, and we expose the intersection matrix to let the user run their own mining algorithms. Custom selection of combinations is possible, even without using MODL. Indeed, MODL can also be used to perform a pre-selection as a starting point for a manual custom based on the biological problematic (e.g. all combinations containing the particular regulator that you are studying, or a systematic study of all combinations of a given order). MODL may need fine tuning of parameters, but we discuss in Supplementary Notes S-11 and S-III ways around this. Approximate itemset miners are comparatively rarer. We hope MODL can be a contribution to the field thanks to its advantages.

### Perspectives

OLOGRAM-MODL is applicable to any problem that can be reduced to quantifying the significance of overlaps between *n* sets of position intervals. Besides epigenetic marks binding sites, associations between sets of regions such as ‘promoters of housekeeping genes’ or ‘Binding sites for the regulator X in the experimental condition Y’ can also be integrated. This is especially important in our ‘big data’ and multi-omics era, as combinations can also be combinations of datasets.

The core idea of MODL, selecting itemsets according to how well they rebuild the ensemble of all itemsets, is precedented (42). The MODL algorithm can be applied to any submodular problem, as the API supports custom error functions. For example, since variable selection in Naive Bayes classifiers is indeed submodular (31), MODL could select combinations that help such a classifier predict the query, as we demonstrate in Results and comparison section. While this is not yet implemented as a core feature, a guided manual example is available in the documentation which can be readily reused on different files. Other custom losses can also be used.

Since the overlaps are considered in terms of *S*(γ) (overlapping base pairs), it is also possible to do a proximity analysis by extending the regions by different values and comparing the significance of enrichment of each. It would be interesting to extend OLOGRAM-MODL to intra-set overlaps, which could be used to model a signal through quasi-Lebesgue integration by converting it into blocks of reads into overlapping regions. Concatenating flags on successive lines could be a way to include temporality. Regions of contact between genomic elements could be represented as sets and their enrichment quantified. The implementation includes notes to facilitate such improvements, and others such as integrating custom shuffles for the user, or remembering regions IDs when shuffling. Indeed, as for *pygtftk* itself, OLOGRAM-MODL was designed to be evolutive and collaborative.

## CONCLUSION

Since human genomic cis-regulation is performed through combinations/complexes of regulators, robustly identifying the statistically enriched ones is an important step in any bioinformatic analysis. The major contribution of this work is the design and implementation of an algorithm to do so, that both mines and evaluates the enrichment for combinations of more than two sets of genomic regions.

*OLOGRAM-MODL* was designed to leverage itemset mining together with a statistical model analysis, and get the strengths of both. A novel optional itemset mining algorithm designed to be resistant to noise and mine for biological complexes, not simply association rules, is proposed. Then, a statistical framework evaluates the enrichment of the combinations using a Negative Binomial model, which is more resolutive than empirical *P*-values while still being immediately understandable. It returns a parsable graphical representation which helps the identification of master regulators, by supporting inexact combinations.

Our approach is validated on artificial data, and shown to be useful in identifying previously neglected regulators associated to FOXA1 and in identidying expected biological complexes in murine promoters. It is implemented as an easy-to-use tool for the scientific community in the *pygtftk* suite, which makes it easy to use in bioinformatic pipelines.

## DATA AVAILABILITY

The complete workflow used in study, along with the full data used, is available as a Snakefile at https://github.com/qferre/ologram-modl_supp_mat.

The approach itself is accessible through the command line interface of the *pygtftk* toolkit, available on Bioconda and from https://github.com/dputhier/pygtftk

## Supplementary Material

lqab114_Supplemental_File
