# Supplementary material for: OLOGRAM-MODL: mining enriched n-wise combinations of genomic features with Monte Carlo and dictionary learning
Source: NAR Genom Bioinform. 2021 Dec 22;3(4):lqab114. doi: 10.1093/nargab/lqab114 (PMC8693575; doi:10.1093/nargab/lqab114)
Supplement: lqab114_Supplemental_File [file lqab114_supplemental_file.pdf]

SUPPLEMENTARY FIGURES

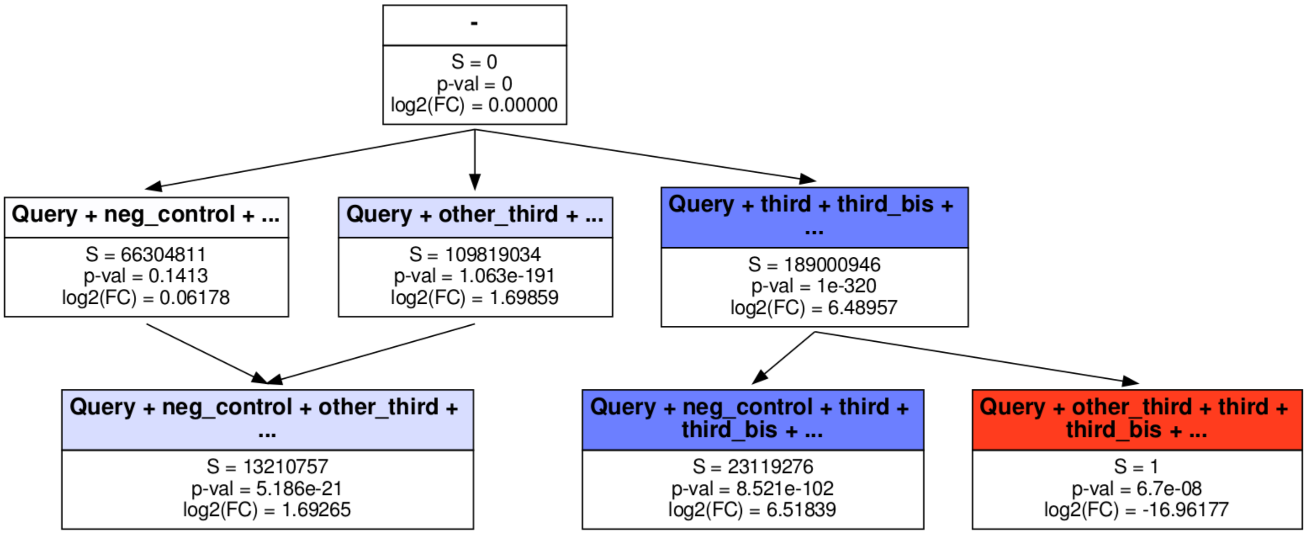

Supplementary Figure 1. OLOGRAM run on the regular artificial data sets. This figure presents a graph-based representation giving the S statistic (number of overlapping base pairs), corresponding Negative Binomial p-value and fold change for each combination. The query was a set of 1,000 artificial regions as described in Supplementary Note S-IV. The combinations are inexact, meaning that an overlap of [Query + neg_control + third] will still count as [Query + neg_control + ...].
OLOGRAM correctly retrieves the associations between sets. The query is shown to be enriched with its subsets. Similarly, the first subset ("third") significantly overlaps its copy "third_bis". The two different subsets ("third" and "other_third") never overlap in the true data, which constitutes a depletion and is marked as such. The negative control, made of random peaks, indeed not overlap the query more than randomly expected. Note that a set C is depleted with the query A but always present with B, and the combination $A+B$ is enriched, the combination $A+B+C$ will be enriched: what matters is what is the difference in enrichment from adding C.
The command line used to run this example was: "gtftk ologram -z -c hg38 -p query.bed –more-bed params.peaks –more-bed-multiple-overlap" where params.peaks is a whitespace separated list of BED files.

Aspect	OLOGRAM-MODL	MULTOVL	GINOM	ChromHMM	TFcoop	LOLA	BEDTOOLS Fisher	HyperGenome browser
Statistical model	Neg. Binom.	Empirical	Bayesian	HMM	Regression	Fisher	Fisher	Empirical
Keeps inter-region distances	Yes	No	No	Yes	No	No	No	Yes
Returns an enrichment value	Yes	Yes	Yes	No	No, but gives a weight	Yes	Yes	Yes
Considers combinations of sets (multiple overlaps)	Yes	Multiplicity only	Yes	Yes	Individual weights only	No	No	No
General speed	Need shuffles	Need shuffles	Costly model	Costly model	Fast	Fast	Fast	Need shuffles
Scales to many sets	Yes, with special representation	Yes, but imprecise	Unlikely	Yes	Yes	Yes	Yes	Yes, but imprecise
Support for any annotation/reference, GTF query customization	Yes	Yes	Difficult	Yes	Yes	Yes	Yes	Yes
Conda installation	Yes	No	No	Yes	No	Yes	Yes	No
Interface	CLI	CLI	None	CLI	CLI	CLI	CLI	Web
P-val precision	Arbitrary	Number of shuffles	Model-dependant	N.A	Model-dependant	Model-dependant	Model-dependant	Number of shuffles
Filtering of combinations or terms	Unsupervised	No	Supervised	No	Supervised	No	No	No
Focus of filtering	Finding complexes, denoising Customizable		Explain the query set		Explain the query set			

Supplementary Table 1. Comparison of the functionalities of OLOGRAM-MODL with existing tools. “Multiplicity only” means the individual members of a combination are not detailed. “Model-dependant” means the model makes questionable statistical assumptions. For MODL’s focus of filtering, “Customizable” means the loss can be customized, which we give an example of in this study through a supervised loss. ChromHMM has a slightly different principle in that it is not designed to give enrichments, but partition the genome by combination.

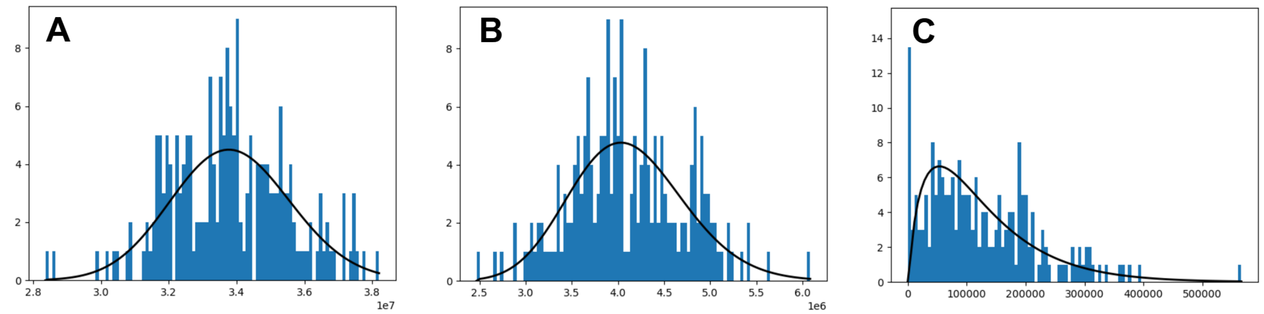

Supplementary Figure 2. Distribution of the number of overlapping base pairs ($S(\gamma)$) in the shufffles, for artificial data of Supplementary Figure 1. Shuffles are made under the null hypothesis that all regions are independent. Negative binomial distributions of same mean and variance as the data are overlaid. We used 200 shuffles.
A is [Query + other_third + ...]
B is [Query + other_third + neg_control + ...]
C is [Query + other_third + third + third_bis + ...]
Even with multiple overlaps, the S statistic still follows Negative Binomial distributions. Longer combinations (such as C here) will be more rarely observed. This supports fitting a Negative Binomial model, as otherwise determining the significance of larger fold changes would require many shuffles. Case C shows an example with a more difficult fitting, as there are few expected overlaps. This leads to a lower precision estimate for the underlying Negative Binomial, but such a low average does not mean much biologically regardless.

**Combination**		**OLOGRAM**			**MULTOVL**			
$E(S_{exp})$ Sets	$S_{obs}(\gamma)$	$E(S_{exp})$	$\sigma(S_{exp})$	p-val	$E(S_{exp})$	$\sigma(S_{exp})$	p-val	Neg. Binom. p-val
A_{21pc}	50695	55610.78	2181.60	0.01046	49267.3	2232.97	0.235703	0.230769
B_{26pc}	63500	55608.89	2149.06	2.117e-4	49267.3	2232.97	0	5.59e-09
$A_{21pc}+B_{26pc}$	3339	13835.87	1206.18	1.17e-39	10899.7	1121.49	0	9.89e-22
Regular negative control	69359776	6.642e+07	2.480e+06	0.1192	6.634e+07	2.619e+06	0.123823	0.125293

Supplementary Table 2. Comparison with MULTOVL. A and B are the artificial coarse datasets (called coarse as they contain a large proportion of the query), while "Regular negative control" is for the negative control in the regular artificial dataset (Supplementary Note S-IV). 1000 shuffles were used in both. The statistic considered is always $S(\gamma)$, the number of base pairs. S_{obs} denotes the true value in the data, while S_{exp} is the value observed in the shuffles. Our p-values differ somewhat, since the expected mean and variance differ due to different shuffling models, but only slightly. In a more typical use case however, with fewer and larger regions, (regular negative control) the p-values match. Importantly, when calculating a p-value using a Negative Binomial and MULTOVL's statistics, this matches MULTOVL p-values for easy cases ($p > 1/n_s$ where n_s is the number of shuffles), which strongly supports our Negative Binomial model.
MULTOVL only gives scores by multiplicity (number of sets overlapping at each position) unlike OLOGRAM which gives the IDs of the sets, which is why we limit ourselves to 2 sets in order to be able to extract information.

Combination		MODL		Exact miners		CL-MAX	
Combi.	True?	Selected at	Score at step 1	Support	Rank	$d = 0.5$	$d = 0.9$
(111100)	Yes	Step 1	-759	2007	18	[3][3*][6][6*]	
(000011)	Yes	Step 2	-799	2632	13	[3][3*][6][6*]	
(110000)	Yes	Step 3	-761	5211	3	[3][6]	[3][3*][6][6*]
(110100)	No	Candidate	-765	2574	14		
(110001)	No	Candidate	-805	723	26		
(111101)	No	Candidate	-809	256	51		
(111110)	No	Candidate	-810	254	52	[6]	
(110011)	No	Candidate	-827	104	53		
(101011)	No	Candidate	-839	70	56		
(011000)	No			2939	11		
(101000)	No			2974	9		
(100100)	No			3001	8		
(010100)	No			2943	10		
(111000)	No			2573	15	[6][6*]	
(110100)	No			2574	14		
(100011)	No			392	35	[6]	
(100010)	No			1066	21	[6*]	
(010010)	No			1052	22	[6*]	
(100001)	No			1132	19	[6*]	[6][6*]
(001000)	No			3688	7		[6][6*]
(001100)	No			2644	12		[6][6*]
(000010)	No			3701	6		[6*]
(000110)	No			767	23		[6]

Supplementary Table 3. Comparison of itemset miners. Comparison of itemsets found by MODL, exact itemset miners (meaning apriori, FP-Growth and LCM) and CL-MAX (approximate itemset miner), on a large artificial matrix. The itemsets used when generating the matrix are represented in mentioned in the "True" column. An uniform noise of 12 % was also applied.

FP-growth and apriori have the same results, as expected. LCM will also return the supports for all the $2^6 = 64$ combinations, since there is both additive and substractive noise: an $A+B+C$ observation can become AB (negative) or $ABCD$ (positive), and no itemset is exactly closed compared to another. As they are exhaustive algorithms, their results are the same. For MODL, we queried 3 atoms, as this corresponds to the number of complexes used when generating the data. The first three atoms returned by MODL correspond to the itemsets defined when generating the data, with the remaining candidates corresponding to noise patterns or compromises. When ordering by support in apriori however, the rank of the true itemsets is much lower.

MODL still gives the correct results even with uniform noise from 0 to 12% (12% is shown here). Furthermore, as expected, the results of an approximate itemset miner such as CL-MAX (based on clustering) are closer to those of MODL: we give CL-MAX's results depending on the discretization threshold (note that $d = 0.9$ was the default parameter), and on how many clusters are requested (number between the brackets) and add an asterisk (ie. "[6*]") for the itemsets returned instead after using their second step (MAFIA algorithm).

However, clustering-based algorithms such as CL-MAX are more vulnerable to a poor choice of hyperparameters, resulting in learning compromises or erroneous itemsets. Results given by CL-MAX also vary heavily depending on random seed. MODL is less vulnerable to this, since in step 2 the selection is based on rebuilding quality, adding irrelevant candidates does not change the first selections.

Combination		Selected by MODL ?		OLOGRAM		GINOM	
Combi.	S_{obs}	Unsupervised	Supervised	Fold change (log2)	p-value	Selected?	Fold change
2	116565	1	4	-0.16616	0.007285	Yes	0.56648
4	38118	2	1	-0.64417	3.866E-07	Yes	0.48758
3	11601	4	2	-0.37221	0.0821	No	No
1	3145	7		-0.7488	0.05463	No	No
2,4	31242	5	6	0.20011	0.1174	No	No
5	22495	8	3	-0.76109	1.176E-05	Yes	0.41661
2,5	18930	3	5	0.13798	0.2523	No	No
4,5	17066			1.11761	6.094E-05	Yes	1.93054
2,4,5	15655	6		2.12929	9.213E-08	No	No

Supplementary Table 4. Comparison with GINOM. We also compare OLOGRAM with GINOM on the demonstration data used in their paper and compare it with their reference results, due to GINOM's unwieldiness. See Supplementary Note S-IV for the meanings of the query and the ID numbers of the sets. S_obs gives the number of base pairs on which this combination is observed to be overlapping with the query (but not in the genome in general, e.g. the set $\{2\}$ overlaps with the query only on 116K base pairs, but covers more than that). The fold changes estimated by GINOM and OLOGRAM are correlated, but their values are slightly different. However, OLOGRAM's fold changes are confirmed by comparison with MULTOVL (Supplementary Table 2). MODL is run on the matrix of intersection described in Figure 2 (1 line per intersection, not per basepair).
Our selection is made differently, though. We give the ranking by order of selection in MODL for the combinations. When pushing the number of combinations required, we get some similarities. Differences can be chalked up to different selection criterion, as we discuss in the main text. Unsupervised MODL is trying to rebuild the original data matrix, not find combinations that best explain the query. For example, while MODL does not select the $\{4,5\}$ combination, it does select the $\{2,4,5\}$ one, which represents 91% of the true base pairs of the $\{4,5\}$ combination. Our analysis took about a minute, less the 20 min advertised for GINOM.
To perform a relevant comparison, we also ran a supervised version of MODL using a custom error function. Here, the error to be minimized is equal to $-F$ where F is the F1-score of a Naive Bayes Gaussian classifier trying to predict the presence of the query region. At each step, features are added to the data given to the classifier, by selecting at each step the new feature that best reduces the error. The presence or absence of each combination is one-hot encoded as a variable (1 means presence). Using a subsampling to get a ratio between the classes "0" and 1 (0 means query is absent, 1 present) of 30:1 and using a weight of 30 for class "1". Note that the subsampling can introduce result variability. Although some necessary assumptions for Naive Bayes are not satisfied (namely, the features are not independent), this is not a problem if the correct class is still more likely than the incorrect one (1). Here, $\{4\}$ is selected first, even though it's not the most abundant, and GINOM agrees. The rest of the selection has a stronger emphasis on set 5, and combinations containing it, as well as combinations containing set 2. To further explain the differences, keep in mind that Naive Bayes classifiers are comparatively not very efficient (even the final classifier with many features still returns many false positives), that the most discriminant features may not be the most abundant (in contrast to unsupervised MODL) and that the most enriched features (as per OLOGRAM and GINOM) may not be turn out to be the most useful predictors (as shown by supervised MODL). Indeed, $\{2,4\}$ being selected before $\{4,5\}$ makes sense since a higher proportion of occurrences of $\{2,4\}$ are overlapping with the query. Results could be improved with further parameter improvement, but the principle is the same. In this analysis, GINOM's selection seems to be equivalent to selecting the lowest OLOGRAM p-values, but mysteriously stopping at order 2. As such, they do not highlight full complexes like $\{2,4,5\}$.

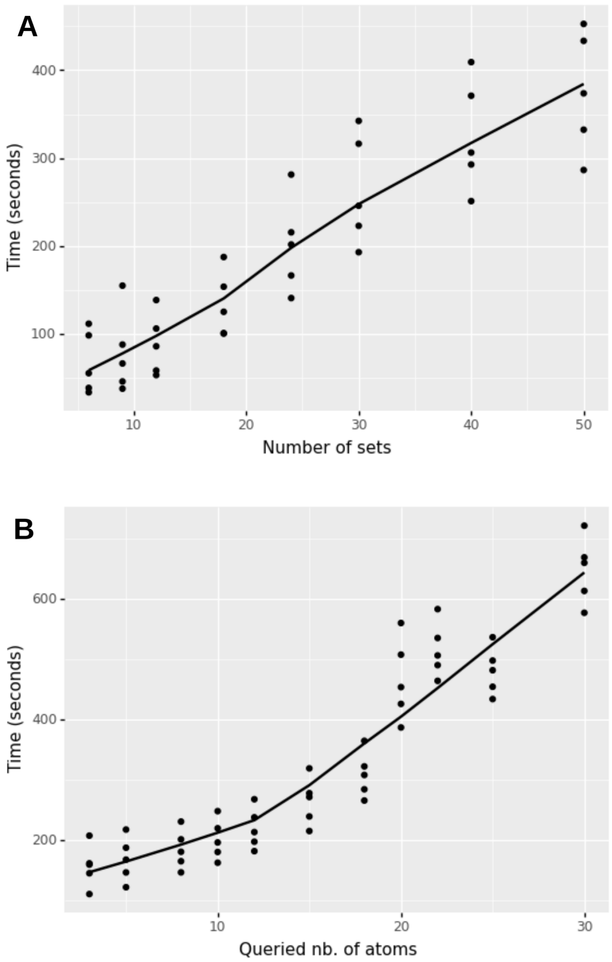

Supplementary Figure 3. Time benchmark of the MODL algorithm. Time cost in seconds, using 8 CPU cores at 2.6 Ghz each. Done on a matrix with 20,000 rows, 50% added noise with the same groups as the usual artificial matrices (AB, ABCD, EF). (A) Scaling with the number of sets (columns) in the matrix. 8 queried atoms. (B) Scaling with the final number of queried atoms. 8 sets in the matrix. MODL scales mostly linearly with the number of sets, but semi-quadratically with the number of queried atoms. Late convergence due to a high α is responsible for most of that time cost for larger matrices. Total time remains on the order of minutes in most cases.

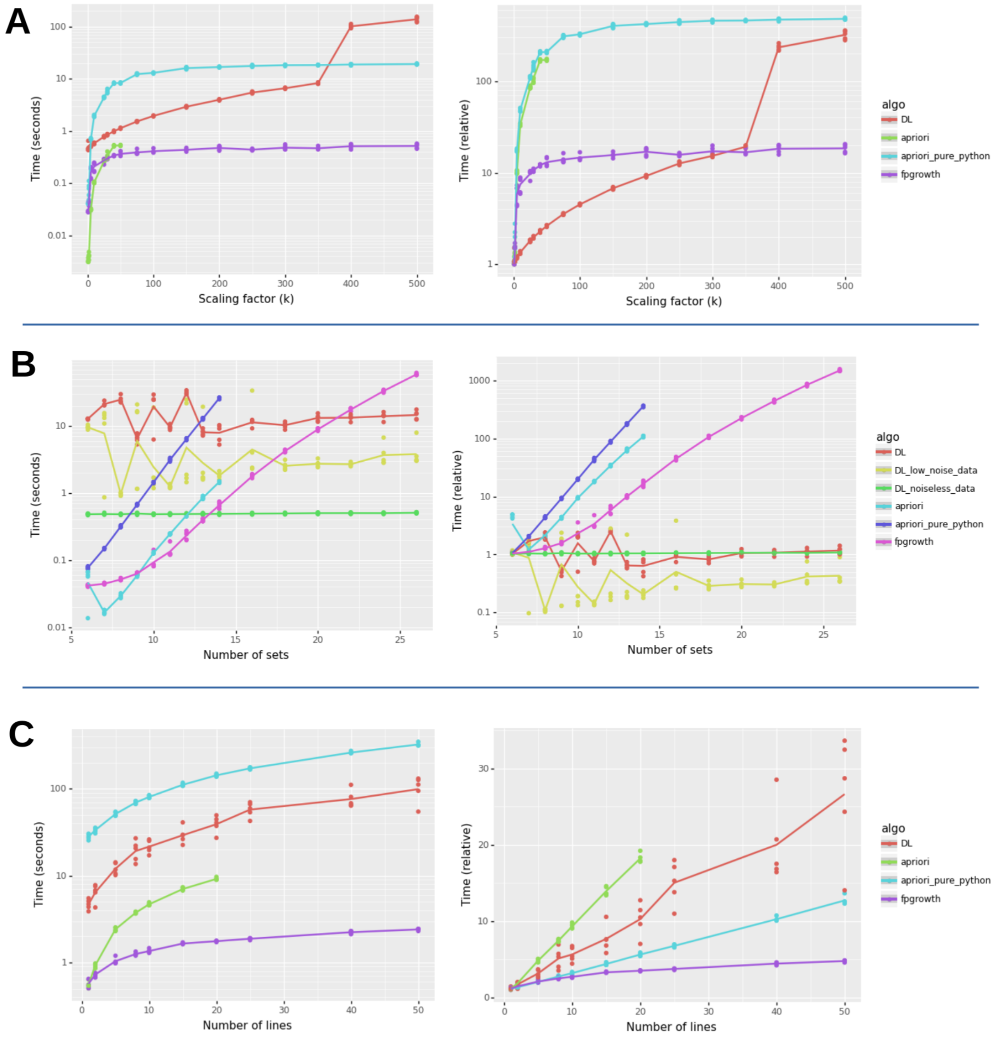

Supplementary Figure 4. Comparison of the scaling of dictionary learning (ie. DL, elementary operation of MODL). Using a single thread on a 2.6 Ghz CPU. Apriori "pure Python" is a Python implementation, the other apriori and FP-growth are the *mlxtend* implementation, DL is the *Scikit-Learn* Python implementation. The left figures gives the absolute time, and the right figures give the times relative to the smallest for this algorithm (to show asymptotic scaling and erase the overhead multiplier due to differences of implementation). (A) a scaling factor of F means DL was instructed to learn F atoms, and apriori and FP-growth had a minimum support of $\frac{1}{F}$. The matrix had 5,000 rows and 13 sets. α for the DL was 0.5. 50% noise was used. (B) Scaling with the number of sets (columns) and a matrix of 5,000 rows. Minimum support for apriori was 0.01, α for DL was 0. DL was trying to learn 120 atoms. Standard noise is 50%, low noise is 1%. (C) Scaling with the number of lines (in thousands). Noise was 50%, minimum support for apriori and fpgrowth was 0.01. The matrix had 15 columns. DL had an α of 0 and learned 120 atoms.

DL scales linearly with the number of sets, while apriori scales exponentially and FP-growth scales quadratically (2). Apriori and FP-growth will scale more than linearly with min_support, until they reach a plateau where the minimum support is so low that all 2^k combinations are considered. On the other hand, DL scales linearly with the number of queried atoms to be learned. Some points on the curves are missing, which is due to implementations used having an impractical RAM or time cost for these parameters, so we stopped before reaching those points. The most peculiar behavior of DL is that, depending on the entropy of the data, it can scale differently. If early convergence cannot be reached, the time cost may be higher for lower-sized data (seen in figure B). But within each sub-slope (early convergence or not) the scaling is linear. DL cost increases faster once early convergence is no longer possible (seen in figure A). When using less noisy data (so the data has less entropy, meaning less information, seen in figure B) DL converges faster and takes less time.

Some scalings are not displayed here. K-means clustering (used in algorithms such as CL-MAX, with Llyod's algorithm) scales mostly linearly with data size, number of sets and number of queried clusters, but it can be superpolynomial in the worst case (3): this is equivalent-to-worse than DL's scaling. GINOM's scaling involves stepwise selection: it is conceptually similar to MODL's step 2 (evaluate all possible additions and carry the best into the next step, see ref. 4) but MODL's advantage is that it does not consider all 2^k candidates, thanks the selection via matrix factoring in step 1.

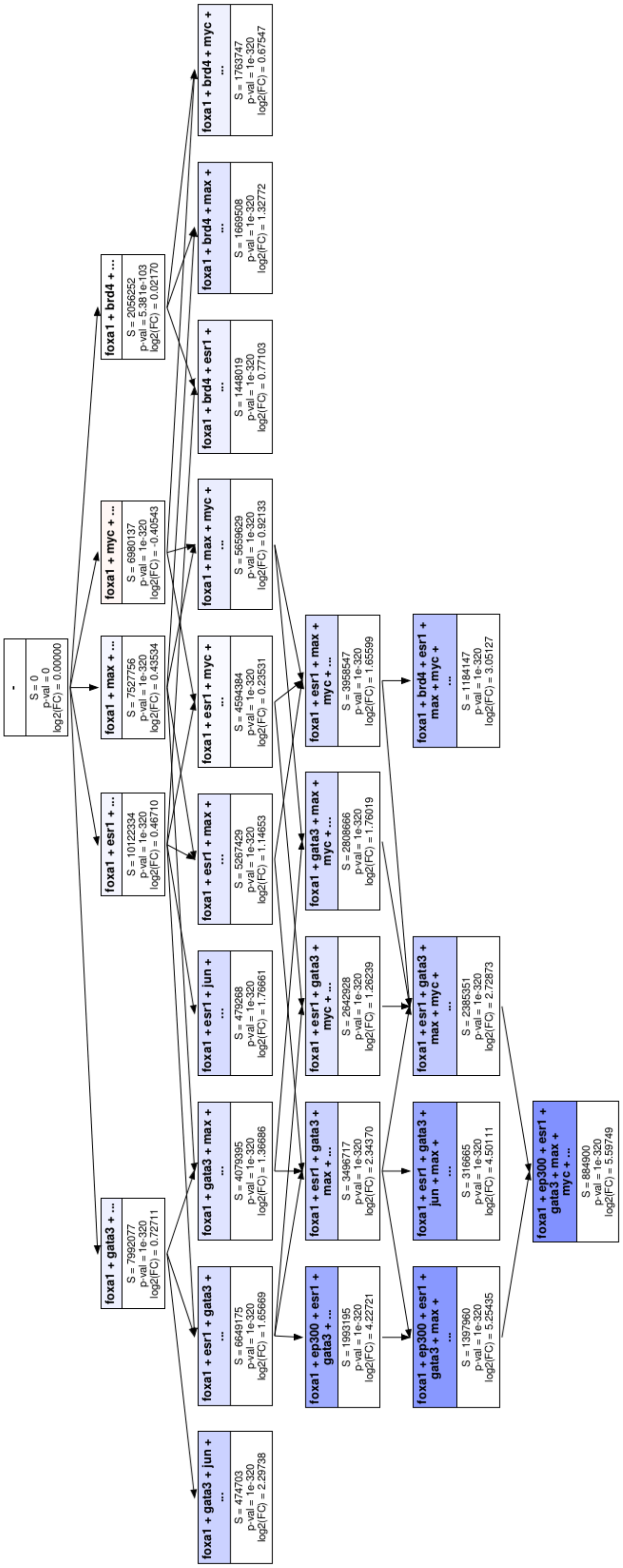

Supplementary Figure 5. Tree of combinations for the FOXA1 analysis in MCF7 but restricted to combinations selected by MODL. MODL selects atoms that best help rebuild the matrix of original overlaps. Rarer but more enriched factors such as EP300, are found with their usual correlators and not alone. MAX and MYC, more frequent, are found more here. We can see that MODL selects relatively closed itemsets.

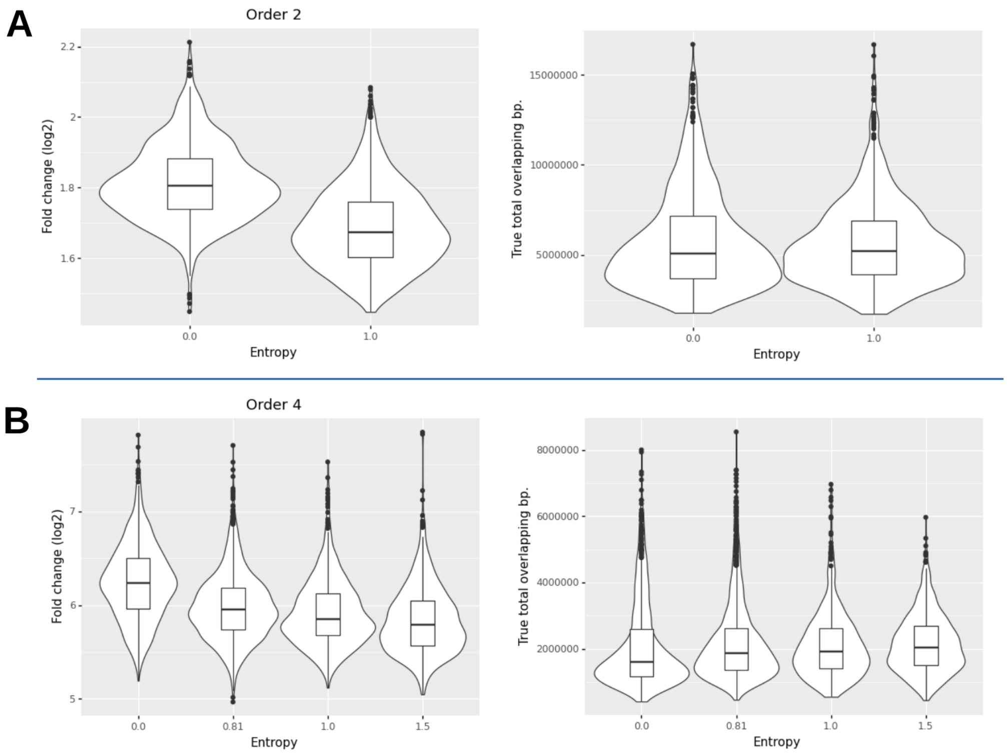

Supplementary Figure 6. Enrichment of sc-ATAC-seq cell combinations by entropy. We randomly selected 75 cells from three different superclusters, based on the classification on the Signac vignette: a "CD14" supercluster, a "CD4 and CD8" supercluster, and a "pre-lymphocyte B" supercluster. The shuffles were not restricted, and MODL is also not used here. We then consider the enrichment for each possible combination γ depending on its heterogeneity (Shannon entropy $H(\gamma)$). For example, consider the combination $A+B+C$ where all three are sc-ATAC-seq sites from CD4 cells: it is homogeneous and has an entropy of 0. However, if D and E are sites for respectively a CD14 and pre-B cell (different superclusters), the combination $A+D+E$ has a high entropy. We expect that heterogeneous combinations (with higher entropy) are less enriched since the open genomic sites of cells of different superclusters would be less similar than between the same supercluster. As usual, we compare one order at a time: (A) 2-wise combinations with 2-wise combinations, and (B) 4-wise with 4-wise.
Homogeneous combinations indeed have higher enrichment (about 1.5 to 2x higher). However, this difference is small, as there are very high individual variations between the cells even within the same supercluster. This is confirmed by simply looking at the true $S(\gamma)$. For higher orders, we instead focus on comparing between $H(\gamma)=0$ and $H(\gamma)>0$, since in the latter cases we are different supercluster compositions may have the same entropy, which would complicate the interpretation, but only pure combinations have zero entropy. This shows that similar cells have similar patterns of chromatin openness, although individual variations are also an important contributing factor.

Group	File path	Description
Plugin	pygtftk/plugins/ologram.py	*Root file.* Parses the arguments and calls the other functions.
Utility	docs/source/ologram.rst	Documentation source.
Root	pygtftk/stats/intersect/overlap_stats_shuffling.py	*Main function.* Called directly by the *ologram.py* plugin. All other functions calls are descended from this one.
Root	pygtftk/stats/intersect/overlap_stats_compute.py	Functions to compute overlap statistics on (shuffled) region sets.
Algorithm	pygtftk/stats/intersect/create_shuffles.pyx	Shuffle BED files and generate new "fake" BED files.
Algorithm	pygtftk/stats/intersect/overlap/overlap_regions.pyx	Compute the overlaps between two sets of genomic regions, supporting multiple overlaps.
Utility	pygtftk/stats/intersect/read_bed/read_bed_as_list.pyx	Turn a BED file into a list of intervals.
Utility	pygtftk/stats/intersect/read_bed/exclude.cpp	Exclude certain regions from a set to create concatenated sub-chromosomes.
Utility	pygtftk/stats/multiprocessing/multiproc.pyx	Helper functions and structures for multiprocessing.
Statistics	pygtftk/stats/negbin_fit.py	Utility functions relative to the negative binomial distribution, including verifying its good fit.
MODL	pygtftk/stats/intersect/modl/dict_learning.py	Contains the MODL algorithm, an itemset mining algorithm described in this paper.
MODL	pygtftk/stats/intersect/modl/subroutines.py	Subroutines of the MODL algorithm. Those are pure functions and can be used independently.
Utility	pygtftk/stats/intersect/modl/tree.py	A graph-based representation of combinations of elements.
Plugin	pygtftk/plugins/ologram_merge_runs.py	Merge a set of OLOGRAM runs into a single run and recalculates statistics based on it.
Plugin	pygtftk/plugins/ologram_merge_stats.py	Merge a set of OLOGRAM outputs calculated on different queries into a single output, preserving labels. Build a heatmap from the results.
Plugin	pygtftk/plugins/ologram_modl_treeify.py	Turns a result of OLOGRAM-MODL multiple overlap (tsv file) in a tree for easier visualisation.

Supplementary Table 5. OLOGRAM-MODL files. Detailed list of the source code files of OLOGRAM-MODL, with their roles. All paths are relative to the root of the *pygtftk* Git repository. The "Plugin" group designates plugins that can be called directly from the *pygtftk* command line. A file extension of *pyx* designates a Cython file, *py* a Python file, and *cpp* a C++ file.

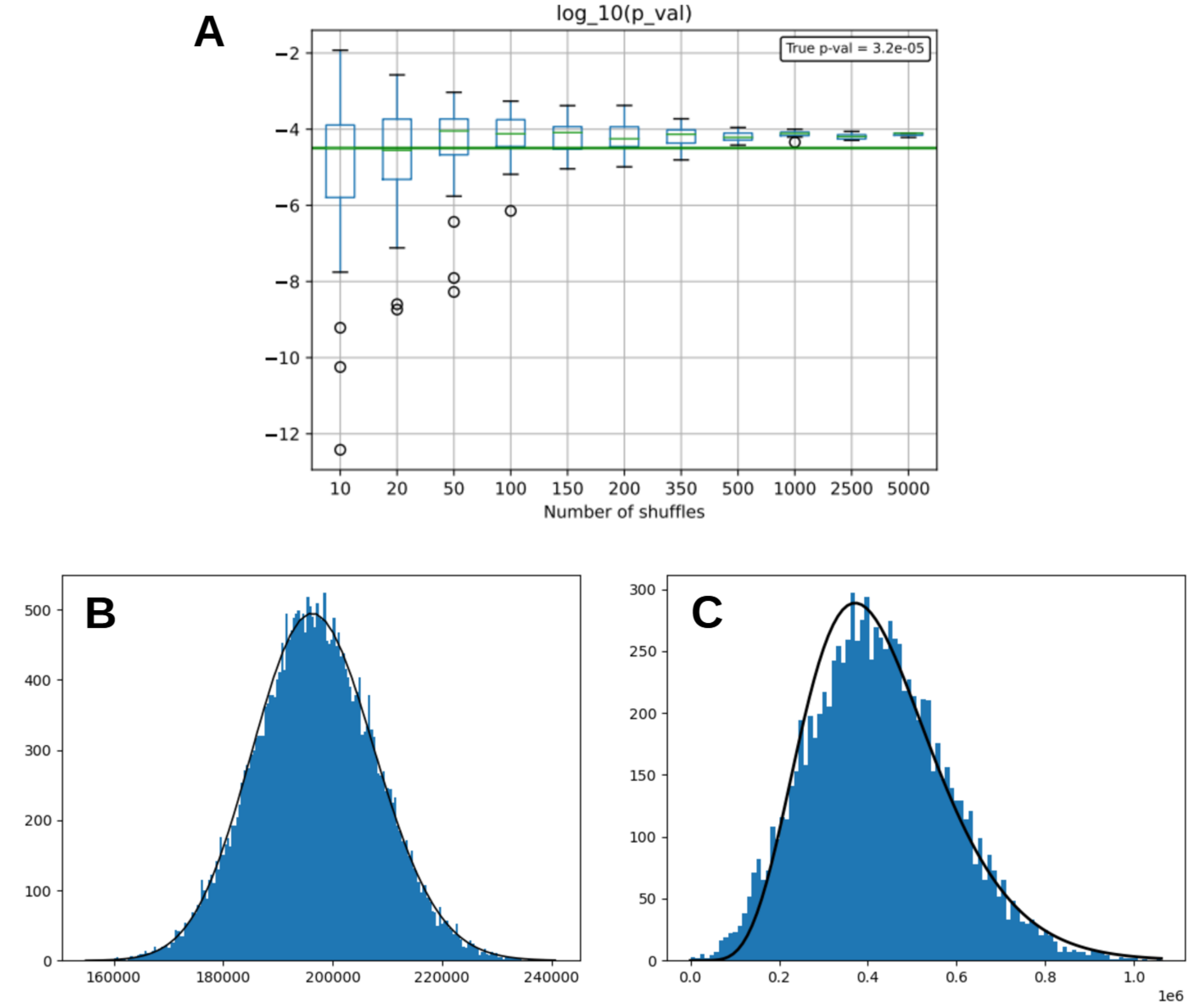

Supplementary Figure 7. Precision of the fitting in the distribution's tails. The combination considered is simply the pairwise combination of $\{A+B\}$ on the "finesse" artificial data. We plot the values of S (total nb. of base pairs on which the combination is observed) in the shuffles.
(A) presents the estimated p-value, compared to the "true p-value", that is to say an empirical p-value calculated with 1 million shuffles. The precision is very acceptable at the very significant level (10^{-5}), but there is a slight overestimation in the tails, the causes of which are expanded upon in Discussion. In summary, it is due to the model being valid only asymptotically.
(B) presents the histogram of the S values in the shuffles performed for (A). The superimposed black line is the probability mass function of a Negative Binomial distribution with the same mean and variance as the data. The empirical values of S are visibly very close to the Neg. Binom. fitted distribution, with, with only small differences appearing in the extreme tails.
(C) is not related to A and B. It is instead an extreme example of the distribution of S in the shuffles, where only 20 regions are present per chromosome. This results in a small number of inter-region distances to be shuffled, making the Neg. Binom. approximation not good enough for the underlying Beta Binomial.

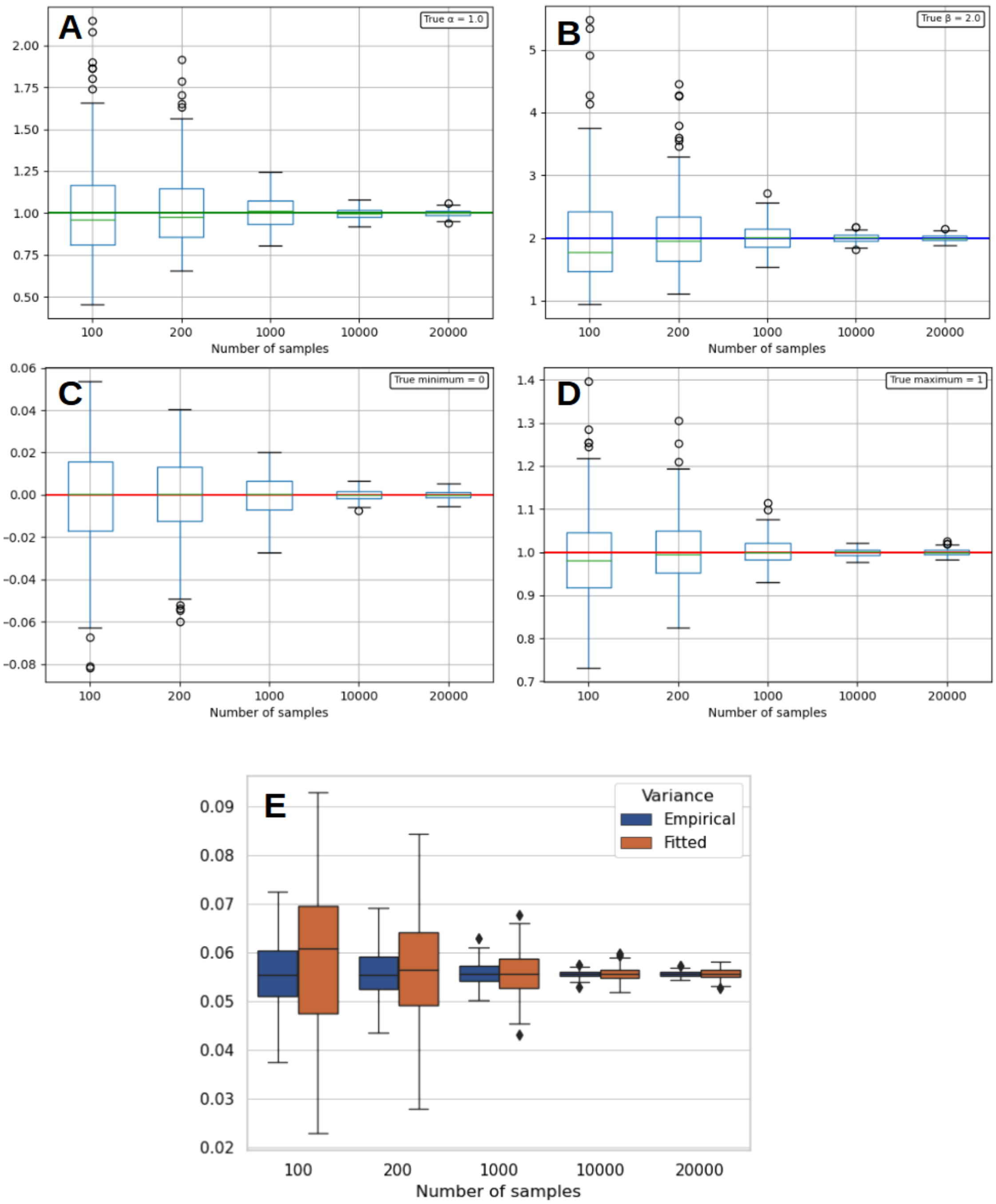

Supplementary Figure 8. Beta distribution's poor fitting. This is an illustrative figure presenting the difficulties of fitting a Beta distribution. Samples are taken from a four-parameter Beta distribution of $\text{Beta}(\alpha = 1, \beta = 0, a = 0, c = 1)$. Recall that α and β are the shape parameters, and that a and c are the scaling parameters giving respectively the minimum and maximum of the possible range of values. Then, the parameters are estimated from the samples themselves using method-of-moments fitting. It can be seen that at 200 data points (corresponding to the usual number of shuffles in OLOGRAM), the estimates for the parameters are very imprecise. This is considerably worse than simply fitting a Negative Binomial: in the subfigure (E), we compare the theoretical variance against the empirical variance. The "theoretical variance" is the expected variance in the samples, calculated using the estimations for the model parameters gained through the method-of-moments fitting. The empirical variance is simply the variance in the generated samples. We can see that the Beta fitting results in a poorer estimation of the true variance. By extension, we deduce that in a set of samples that imperfectly resembles a Negative Binomial distribution, the Beta-fitted tails (and by extension the p-values) would be estimated with even more errors than merely using a Neg. Binom. fitted based on the empirical variance.

SUPPLEMENTARY NOTES

S-I Pre-processing of matrices before MODL

Definition 1. *The abundance of a row r in a given matrix X is the number of rows in X which are exactly equal to r. It is noted $a_X(r)$.*

Definition 2. *Let $s(X)$ be the smallest abundance in X among the abundances for all the unique rows of X. The smothered version of the matrix X is the matrix $\psi(X)$. For each unique row x of X, $a_{\psi(X)}(x) = \sqrt{\frac{a_X(x)}{\nu}}$, where ν is the highest of either $s(X)$ or the abundance threshold τ. Row order is unimportant.*

After smothering, X is reduced to one elementary repetition of itself to save computing time. The default abundance threshold is $\tau = 1\mathrm{e}{-4}$, combinations rarer than this are ignored. The abundance threshold τ helps reducing the time cost of MODL, analogous to the minimum support used in other itemset miners to limit the exponential complexity of the problem.

The use of the square root of the abundances gives more weight to the rarest combinations, instead of simply focusing on an even better reconstruction of frequent combinations. It is the smothered version of X that is passed to the MODL algorithm, not X itself.

S-II Additional information on MODL's library creation step

The reconstructions are repeated on a 3-fold cross-sampling (rotating 2/3 of data) to increase result variety, as random initialization can result in different reconstructions. Coordinate descent with LASSO is used for the fitting with 200 iterations, where negative atoms are disallowed to allow later interpretation. The more widespread Least-angle regression (LARS) fitting algorithm is known to select wrong features when the features are correlated (5) and as such is is only used as a fallback if LASSO fails to converge.

A higher number of atoms in the learned dictionary would result in more precise reconstructions, but lessen the need to learn itemsets instead of individual components or simply unique rows (*i.e. words*). Conversely, fewer atoms will result in learning compromise atoms. Although this is the point of the approach and grants resistance to noise (both false positive and negatives), too much compromise can result in learning potential correlation groups such as using only an atom (111) to reconstruct the words (100) or (011). As a trade-off between having variety and the effects just mentioned, the number of atoms in the learned dictionary is by default $2 \times q$.

As too low values can hinder convergence, the sparsity constraint α used in those reconstructions begins at $1/k$ where k is the number of sets (ie. number of columns of X). Using increasing steps avoids lingering at high α, where results can be redundant. This can however result in the algorithm spending more time at high α, which will result in longer words and compromise words (as discussed above), which will then have higher total usage (see below). As such, the sparsity constraints used in this step can be overriden as a parameter in the API, if one wishes to produce more shorter words by spending more steps at low α.

After each factorization, each atom v is binarized into a discretized vector $\mathrm{Discr}(v)$, and saved along with its total usag (the multiplicative coefficient associated to it in the reconstructions, see section 2.2.1 for the precise definition). For each value v_i in the atom v (v is a vector), the corresponding value in the discretized vector $\mathrm{Discr}(v)$ is :

$$\mathrm{Discr}(v_i) = \begin{cases} 1 & \text{if } v_i > \frac{1}{k^2} \text{ and } v_i > d * \max(v) \\ 0 & \text{else.} \end{cases}$$

The d is called the discretization threshold, with a default value of 0. The use of custom discretization thresholds, as well as the manual α selection discussed above, would be key to stop MODL from learning too many "compromise words" as discussed above and learn words that are closer to classical association rules, which can then be sorted in step 2.

Finally, once all reconstructions are done, a library Λ of candidate atoms is obtained. In order to save time, only the top $3 * q$ atoms ordered by their summed usage across all reconstructions are kept before passing Λ to the next step. This will also discard leftover atoms with low usage. An optional filtering by atom length is possible.

S-III Additional information on MODL's atom selection step

The sparsity controller α tends to emphasize the longest atoms: this impact is reduced by projecting each $\lambda \in S$ on the surface of the 1-unit ball ($\|\lambda\|_2 = 1$). To ensure no two atoms have the exact same dot product with a given word, a small jitter of $\frac{\sqrt{i}}{10^4}$ is added to each value of the i-th atom. S is sorted in lexicographic order. As the set of atoms usually has some degree of degeneracy (similar atoms), the sparse coder used is LASSO-LARS. Coordinate Descent does not handle it well, but LARS tends to drop correlated regressors, which is a strength here. In any case, the process does not compare the usage of each atom, only the quality of the reconstruction.

The sparsity controlling parameter α on both the coder's LASSO and the d_1 error is nonzero, in order to encourage adding (11) to the dictionary even if (01) and (10) are already present, as that would bring an improvement by using only one atom. Manually computing the Manhattan error in the evaluation instead of Euclidian penalizes rebuilding both (01) and (10) as $(\frac{1}{2}\frac{1}{2})$. A relatively high $\alpha = \frac{1}{\sqrt{k}}$ (capped at 0.5) is used by default, but can be changed. This helps convergence and emphasizes using as few words as possible to get closed itemsets, instead of focusing on improving the rebuilding of frequent combinations.

In case of a tie, the first atom is selected. This is a greedy algorithm, in that it makes the locally optimal choice at each iteration. The raised solution is optimal if the optimization problem is the maximization of a submodular function (diminishing returns when adding new elements). A set function f is submodular if:

$$\forall X, Y \subseteq \Omega \text{ with } X \subseteq Y \text{ and every } x \in \Omega \setminus Y \text{, we have}$$
$$f(X \cup \{x\}) - f(X) \geq f(Y \cup \{x\}) - f(Y)$$

Then the use of a greedy algorithm to maximize f will produce a solution within a factor of $1 - 1/e$, which is the best possible approximation (6).

Proposition 1. *The problem of finding* $S^* = \arg\max_S f(S)$ *admits a good submodular approximation.*

Proof sketch. Consider the gain made by adding λ so a set S, and consider S' a set of atoms such that $S \subseteq S'$. Assuming the sparse encoder optimizes correctly, the encoding cannot be made worse by adding more atoms to choose from in the dictionary: if useless, they will simply be ignored. So $f(S) \leq f(S')$, and f is monotonous.

Furthermore, adding redundant atoms does not improve the reconstruction further, resulting in diminishing returns: for each row (*i.e.* word) $x \in X$ to be rebuilt, the solution found is a linear combination of the atoms in the dictionary. Adding a new atom λ to this dictionary will only improve the reconstruction depending on the coefficient given to the new atom, which is turn depends on how much error the previous dictionary made on this word x that the new atom was brought to correct. Indeed, the improvement that a candidate λ can bring is bounded by the difference in the projections of x on the subspaces defined by V_t and by $V_t \cup \lambda$ (7). While this approximate submodularity of f is valid only up to a margin dependent on the incoherence of the matrix of candidates, in practice even coherent ones result in good approximations (7, 8). This is even more true due to α which penalizes the use of too many atoms to rebuild x. □

Perspectives In order to improve the scaling with the number of queried atoms, it would be possible in this test to not always test all candidates, but only the children combinations (see definition in section 2.1.1) of the already selected combinations/atoms, assuming that if an atom A is better than an atom B, then its best parent is better than the best parent of B, proceeding until an optimum is reached.

If the problem is not submodular, there is a potential alternative (but costly) method. After adding the best word, the least useful word can be removed after each iteration. The algorithm would then continue until the maximum number of steps is reached, or where the word being added and removed are the same ("convergence"). This would be analogous to gradient descent.

S-IV Data

Artificial and comparison datasets and tools These datasets are used to demonstrate and quantify the behavior of our algorithms.

1. **Noisy intersections matrices**: an artificial overlap matrix whose unique rows are representation of $A+B$, $A+B+C+D$ or $E+F$. This equates to row vectors of respectively $(110000), (111100), (000011)$. A *NOT* is then applied to each element (turning a 0 into 1, and 1 into 0) with a probability p_N to represent noise. We use a matrix with a large number of rows to perform a large-scale experiment and ensure the results are not due to random chance. The itemsets selected represent the biologically interesting cases, with a diversity of complexes and two overlapping ones.

2. **Artificial BEDs of regions**: a query set of 1,000 artificial genomic regions of length 200,000 has been generated in in the *hg38* genome and compared against the following sets: (A) a random third of the regions of the query set, (B) a copy of this A set, (C) a different third of the query set that does not overlap with the selection in A, and (D) a negative control made of other random peaks. This is termed the *regular* artificial data in the following text. We also prepare a different, *coarse* artificial data set with a query set of 10,000 regions, 250bp length each, in an artificial genome of one chromosome of 1Mbp. The A set takes the first 21% of query, and B takes the last 26% of query (so we would expect A and B to overlap less than by chance). It is called coarse as the subsets contain

a large proportion of the query. Finally, a third dataset called “finesse” is prepared by drawing regions from a genome which has 5 chromosomes of 100M base pairs. The set *A* contains 10,000 regions of length 1,000 bp. *B* is made of 10,000 other regions of the same size drawn independently plus 51 regions copied from *A*.

3. Additionally, when comparing against GINOM, we use their demonstration dataset. The query consists of somatic L1 retrotranspositions identified in lung cancer. The reference sets consist of, with their number: (1) RefSeq human promoters long of 2000 bp, (2) RefSeq gene bodies, (3) binding sites for H3K4me3, (4) for H3K36me3 and (5) for H3K79me2. In the absence of explanations, alphabetical order was assumed for the reference numbering. Binding sites are broad ChIP-Seq peaks from the A549 cell line. All regions are in the *hg19* genome. More details are available from 9.

When comparing our tool against other algorithms, we used the *mlxtend* implementations of FP-growth and apriori (10). For LCM (11) we used the *SPMF* implementation (12). We used the version 1.3 (2018) of MULTOVL. Other tools' sources are referenced in the text directly when relevant.

Biological datasets We use these datasets to demonstrate the utility of OLOGRAM-MODL on biological problems. The ground truth is not known beforehand, instead results are interpreted biologically.

1. **Transcription Factor Binding Sites data**: selected binding regions from ReMap 2018 data (13) for the transcription factors FOXA1, BRD4, EP300, ESR1, GATA3, JUN, MAX, MED1 and MYC in the *hg38* human genome assembly, for the *MCF7* breast cancer cell line.

2. **sc-ATAC-seq data**: single cell ATAC-seq data and estimated cellular clusters for human peripheral blood mononuclear cells was drawn from the vignette of the Signac package (14).

3. **Murine data**: murine ChIP-seq data for the TFs: NANOG, KLF4, POU5F1, SOX2, STAT1, STAT6, SMC1A, IRF1, CTCF, RAD21, SMC3, IRF9 was drawn from ChIP-atlas (15), and the National Bioscience Database Center, in the *mm10* genome. Murine promoters are estimated by extending estimated murine TSS by 1,000 base pairs to the left, where the TSS were drawn from refTSS (16).

REFERENCES

[1] Rish, I. (January, 2001) An Empirical Study of the Naïve Bayes Classifier. *IJCAI 2001 Work Empir Methods Artif Intell,* **3**.

[2] Anand, H. S. and Vinodchandra, S. S. (April, 2018) Association rule mining using treap. *International Journal of Machine Learning and Cybernetics,* **9**(4), 589–597.

[3] Arthur, D. and Vassilvitskii, S. (June, 2006) How slow is the *k*-means method?. In *Proceedings of the twenty-second annual symposium on Computational geometry* New York, NY, USA: Association for Computing Machinery SCG '06 pp. 144–153.

[4] Zhang, Z. (April, 2016) Variable selection with stepwise and best subset approaches. *Annals of Translational Medicine,* **4**(7).

[5] Efron, B., Hastie, T., Johnstone, I., and Tibshirani, R. (April, 2004) Least angle regression. *Annals of Statistics,* **32**(2), 407–499.

[6] Feige, U. (1998) A Threshold of ln n for Approximating Set Cover. *Journal of the Acm,* **45**, 314–318.

[7] Krause, A. and Cevher, V. (June, 2010) Submodular dictionary selection for sparse representation. In *Proceedings of the 27th International Conference on International Conference on Machine Learning* Madison, WI, USA: Omnipress ICML'10 pp. 567–574.

[8] Das, A. and Kempe, D. (February, 2011) Submodular meets Spectral: Greedy Algorithms for Subset Selection, Sparse Approximation and Dictionary Selection. *International Conference on Machine Learning,* arXiv: 1102.3975.

[9] Bryner, D., Criscione, S., Leith, A., Huynh, Q., Huffer, F., and Neretti, N. (June, 2017) GINOM: A statistical framework for assessing interval overlap of multiple genomic features. *PLOS Computational Biology,* **13**(6), e1005586.

[10] Raschka, S. (April, 2018) MLxtend: Providing machine learning and data science utilities and extensions to Python's scientific computing stack. *Journal of Open Source Software,* **3**(24), 638.

[11] Uno, T., Kiyomi, M., and Arimura, H. (January, 2004) LCM ver. 2: Efficient Mining Algorithms for Frequent/Closed/Maximal Itemsets.

[12] Fournier-Viger, P., Gomariz, A., Gueniche, T., Soltani, A., Wu, C.-W., and Tseng, V. S. (January, 2014) SPMF: a Java open-source pattern mining library. *The Journal of Machine Learning Research,* **15**(1), 3389–3393.

[13] Chèneby, J., Gheorghe, M., Artufel, M., Mathelier, A., and Ballester, B. (January, 2018) ReMap 2018: an updated atlas of regulatory regions from an integrative analysis of DNA-binding ChIP-seq experiments. *Nucleic Acids Research,* **46**(D1), D267–D275.

[14] Stuart, T., Srivastava, A., Lareau, C., and Satija, R. (November, 2020) Multimodal single-cell chromatin analysis with Signac. *bioRxiv,* p. 2020.11.09.373613.

[15] Shinya, O., Tazro, O., Go, S., Hideki, H., Osamu, O., Yoshihiro, O., Hideya, K., Ryo, N., Jun, S., and Chikara, M. (2018) ChIP-Atlas: a data-mining suite powered by full integration of public ChIP-seq data. *EMBO Rep,*.

[16] Abugessaisa, I., Noguchi, S., Hasegawa, A., Kondo, A., Kawaji, H., Carninci, P., and Kasukawa, T. (June, 2019) refTSS: A Reference Data Set for Human and Mouse Transcription Start Sites. *Journal of Molecular Biology,* **431**(13), 2407–2422.
